# A novel TOX3-WDR5-ABCG2 signaling axis regulates the progression of colorectal cancer by accelerating stem-like traits and chemoresistance

**DOI:** 10.1371/journal.pbio.3002256

**Published:** 2023-09-14

**Authors:** Jiaojiao Hao, Jinsheng Huang, Chunyu Hua, Yan Zuo, Wendan Yu, Xiaojun Wu, Liren Li, Guoqing Xue, Xinyu Wan, Liyuan Ru, Ziyue Guo, Shilong Han, Wuguo Deng, Fei Lin, Wei Guo

**Affiliations:** 1 Institute of Cancer Stem Cells & The First Affiliated Hospital, Dalian Medical University, Dalian, China; 2 Sun Yat-sen University Cancer Center; State Key Laboratory of Oncology in South China, Collaborative Innovation Center of Cancer Medicine, Guangzhou, China; 3 Department of Oncology, Guangdong Provincial Hospital of Integrated Traditional Chinese and Western Medicine; The Affiliated Nanhai Hospital of Traditional Chinese Medicine of Jinan University, Foshan, China; B.C. Cancer Agency, CANADA

## Abstract

The eradication of cancer stem cells (CSCs) with drug resistance confers the probability of local tumor control after chemotherapy or targeted therapy. As the main drug resistance marker, ABCG2 is also critical for colorectal cancer (CRC) evolution, in particular cancer stem–like traits expansion. Hitherto, the knowledge about the expression regulation of ABCG2, in particular its upstream transcriptional regulatory mechanisms, remains limited in cancer, including CRC. Here, ABCG2 was found to be markedly up–regulated in CRC CSCs (cCSCs) expansion and chemo–resistant CRC tissues and closely associated with CRC recurrence. Mechanistically, TOX3 was identified as a specific transcriptional factor to drive ABCG2 expression and subsequent cCSCs expansion and chemoresistance by binding to −261 to −141 segments of the ABCG2 promoter region. Moreover, we found that TOX3 recruited WDR5 to promote tri–methylation of H3K4 at the ABCG2 promoter in cCSCs, which further confers stem–like traits and chemoresistance to CRC by co–regulating the transcription of ABCG2. In line with this observation, TOX3, WDR5, and ABCG2 showed abnormal activation in chemo–resistant tumor tissues of in situ CRC mouse model and clinical investigation further demonstrated the comprehensive assessment of TOX3, WDR5, and ABCG2 could be a more efficient strategy for survival prediction of CRC patients with recurrence or metastasis. Thus, our study found that TOX3–WDR5/ABCG2 signaling axis plays a critical role in regulating CRC stem–like traits and chemoresistance, and a combination of chemotherapy with WDR5 inhibitors may induce synthetic lethality in ABCG2–deregulated tumors.

## Abstract 2

This study reveals that a novel TOX3-WDR5-H3K4me3/ABCG2 signaling axis is upregulated in colorectal cancer (CRC) stem cells; these proteins show potential as biomarkers for predicting survival of CRC patients, and targeting the pathway may help overcome the challenges of drug tolerance, recurrence and metastasis.

## Introduction

As the most common malignancy of the digestive system, colorectal cancer (CRC) also remains one of the leading causes of cancer-related deaths in both economically developed and developing countries [1], and the pathogenic mechanisms related to CRC initiation and development have always been the hotspots in oncology. However, the enthusiasm generated by recent progress in CRC prevention, diagnosis, and intervention, has been tempered by the recurrence and drug resistance in the clinic, which greatly reduces drug efficacy and worsens patient outcome [2,3]. Cancer stem cells (CSCs) with stem-like characteristic, that is, the ability to self-renew and differentiate into multiple tumor cell types, are generally considered to be the key cells to build for the initiation, propagation, chemoresistance, metastasis, and relapse of different cancers, of course, including CRC [4–6]. Targeting CSC has been considered as a promising anticancer strategy. Therefore, there is an urgent need to further elucidate the molecular signatures affecting colorectal cancer stem cells (cCSCs) evolution, clarify what might confer these molecular signatures and consequently identify new effective intervention targets.

Numerous stem-like traits-associated biomarkers and signaling pathways have been reported to be implicated in cCSCs maintenance and propagation, such as CD133, CD24, CD29, CD44, CD166 (ALCAM), Lgr5, EPCAM, and ABCG2, etc. [7–10]. ABCG2 shows a high physiological expression in multiple tissues, including intestinal epithelium, placenta, blood–brain barrier, and various stem cells [11]. In regard to stem cell research, the high expression of ABCG2, combined with the pump-out function of fluorescent dyes, is employed to identify hematopoietic stem cells, while pluripotent stem cells defined by the so-called “side population (SP)” of progenitor cells actively pumping out fluorescent Hoechst 33342 dye also highly expressed ABCG2 in multiple tissues [12]. As a member of the ABCG subfamily, ABCG2 is a drug transporter with a wide range of substrate specificities, including large molecules with positive and negative charges, and amphiphilic molecules. It was first documented that ABCG2 was overexpressed in drug-selective cell lines of ovarian, lung, breast, colon, and gastric cancer [13], and mediates in vitro resistance to a variety of cytotoxic compounds, including mitoxantrone [14], topology Tican, irinotecan active metabolite (SN-38) [15], etc. Moreover, increasing data indicate that individuals with high ABCG2 expression usually respond poorly to chemotherapy [16], which means that tumors expressing sufficient ABCG2 are more likely to develop drug resistance. In addition, ABCG2 expression was significantly increased in irinotecan-resistant CRC cell lines, and its overexpression was also found in in vivo metastases in patients treated with irinotecan [17]. In general, the overactivation of ABCG2 functions as a critical contributor to drive cancer cell chemoresistance and metastasis. Figuring out the precise upstream regulatory mechanism of ABCG2 in CRC progress, especially in multidrug resistance and stem-like traits maintenance, is of great significance.

As reported [18], chromatin modifying factors play important roles in determining cell functions, for instance, the ability of histone methylation in affecting the stem-like traits and drug resistance of cancer cells through the promotion or suppression of gene transcription has been well established [19,20]. Therefore, it is necessary to further explore the underlying relationship between these chromatin modifiers and cancer stem-like traits-related protein expression. As an essential component of SET/MLL methylase complexes, WDR5 is involved in the mono-, di-, and tri-methylation of histone H3 at the lysine 4 (H3K4), which represents a specific tag for epigenetic transcriptional activation and in turn contributes to tumorigenesis and development in various types of cancer [21–23]. Meanwhile, by directly interacting with the pluripotent transcription factor OCT4, and together with SOX2 and NANOG [24,25], to activate gene expression necessary for pluripotency and self-renewal, WDR5 is also an important component of the core pluripotency transcription network [23]. Therefore, most likely, WDR5 is recruited by some specific transcriptional factors to co-promote the expression of stem-like traits-related or drug-resistant genes in cancer treatment and then affects the prognosis of cancer patients.

TOX3, also known as TNRC9 or CAGF9, contains an HMG-box that can participate in bending and unwinding of DNA and changes in chromatin structure. As a transcriptional co-activator, TOX3 has been reported to interact with the p300/CBP transcriptional complex [26]. TOX3 can also prevent cell death by inducing anti-apoptosis and inhibiting pro-apoptotic transcripts and stimulate transcription by binding to the BCL-2 promoter region [27]. In addition, single-nucleotide polymorphisms (SNPs) of TOX3 have been found to be closely related to breast and lung cancer risk [28,29]. Moreover, single-cell sequencing data [30] have shown that TOX3 acts as one of the 6 novel candidate regulators that produce specific enteroendocrine phenotypes. Recently, it has been shown TOX3 may be an oncogene in CRC and predict DFS of CRC patients [31]. However, the precise function and the related molecular mechanisms of TOX3 in CRC progression, especially in stem-like traits maintenance and drug resistance development, have not yet been defined.

In this study, we provided evidence for ABCG2 as a drug-resistant gene up-regulated in chemo-insensitive cCSCs and CRC tissues through RNA-seq analysis and high-throughput screening and elucidated TOX3 as a pivotal regulatory factor to facilitate cCSCs self-renewal and drug resistance, including chemotherapy and targeted therapy, in vivo and ex vivo, by activating ABCG2 transcription. Mechanistically, WDR5 was identified to be recruited by TOX3 to co-anchor at ABCG2 promoter region and synergistically regulating ABCG2 expression through tri-methylation of H3K4 in cCSCs. WDR5 also showed synergy in cCSC expansion and chemoresistance induction mediated by TOX3. The whole study sets out to provide the probability of developing TOX3-WDR5/ABCG2 signaling axis as the candidate cascade therapeutic targets or biomarkers for adjuvant treatment or survival prediction in CRC patients receiving postoperative chemotherapy or with metastasis.

## Results

### ABCG2 expression is preferentially up-regulated in cCSCs

In order to obtain CRC spheres with self-renewal, chemoresistance, and distinct characteristics of cCSCs, we enriched cCSCs by inducing CRC spheroid formation in the presence of chemotherapeutics (oxaliplatin 1 μg/ml). To confirm the stem-like traits of these spheres, colony formation assay, cell invasion experiments, and detection of cell surface markers were respectively performed. Compared to adherent cells, these spheres showed higher colony-forming ability, cell invasion capability, and cancer stem-like traits-related protein expressions (S1A and S1B Fig). Consistently, higher xenograft tumor formation ability of these CRC spheres than adherent cancer cells was observed in in vivo experiments (S1C Fig), and primary cells obtained from xenografts of CRC spheres displayed stronger colony formation and spherization capacity again in vitro (S1D and S1E Fig). According to the results proven above, we initially concluded that these tumor spheres cultured with oxaliplatin could be defined as cCSCs by exhibiting a series of CSC traits, including great proliferative potential and increased epithelial–mesenchymal transition.

**Fig 1:**
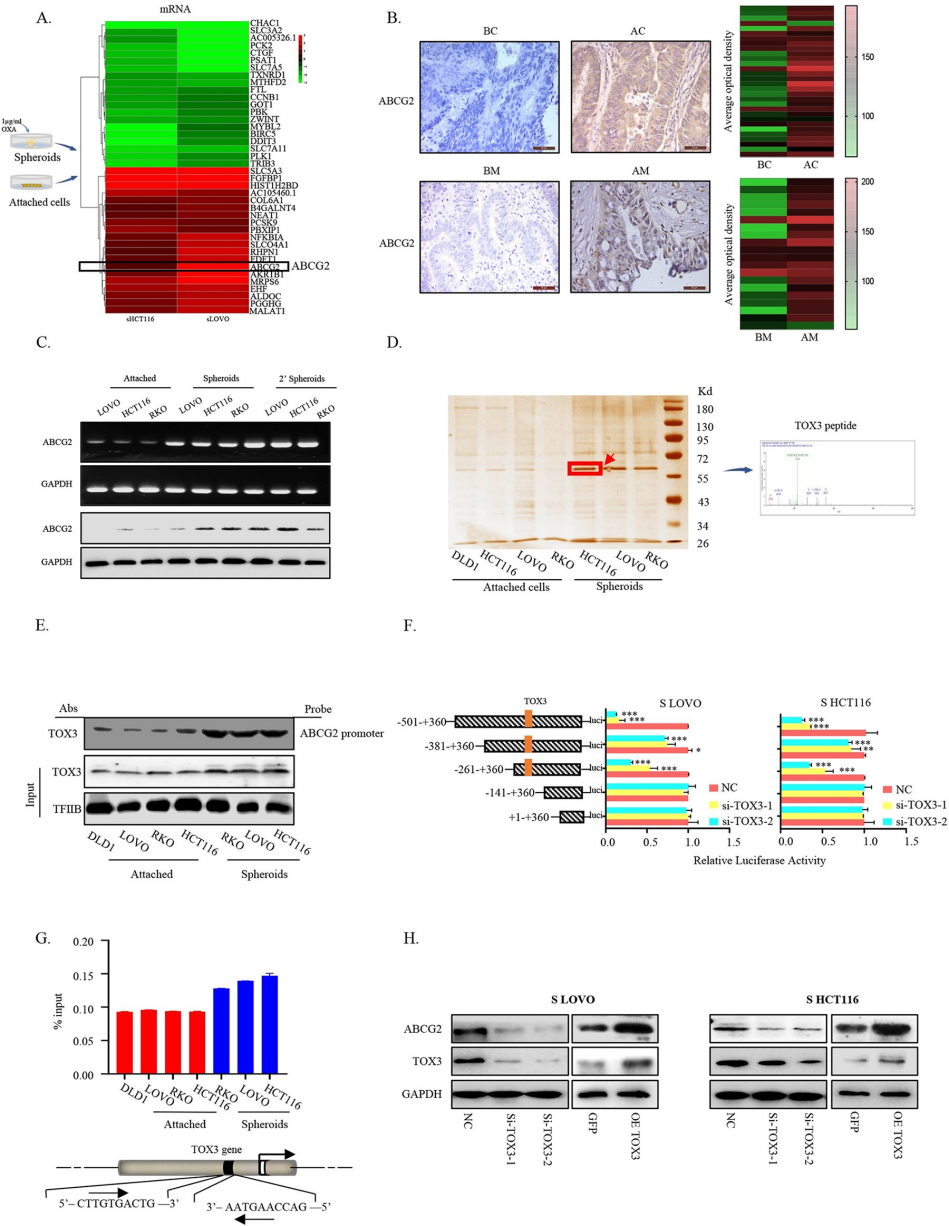
ABCG2 was highly expressed and transcriptionally regulated by TOX3 in cCSCs. (A) Schematic illustration of the experimental approach used to enrich cCSCs and the differentially expressed genes between cCSCs and their counterparts (attached cells cultured without oxaliplatin) from RNA–seq. (B) The representative IHC staining of ABCG2 and the scores of all the IHC staining in tumor tissues of CRC patients, including those BC, recurrent AC, primary tumor BM, and AM. The heat map is a statistical map of the averaged optical density values of the slices stained with ABCG2 antibody from 50 patients with or without chemotherapy (*n* = 30) and with or without metastasis (*n* = 20). (C) Expressions of ABCG2 in 2 successive generations of CRC spheroids and attached cells were examined by RT–PCR and western blot. (D) The streptavidin–biotin DNA pull–down assay was performed to find out the specific proteins which bind to the ABCG2 promoter, and protein bands were visualized by silver staining. The arrow indicates the candidate ABCG2 promoter–binding protein and the corresponding peptide peak analyzed by MS. (E) Western blot assay using an anti–TOX3 antibody for the binding detection of TOX3 to ABCG2 promoter probe after the streptavidin–biotin DNA pull–down assay. (F) Re–attached CRC cells were co–transfected with TOX3 siRNAs, ABCG2 promoter–driven luciferase plasmids, and renilla luciferase reporter plasmids and luciferase activity was finally detected. (G) Chromatin immunoprecipitation assays were used to detect the binding of TOX3 protein to ABCG2 promoter in the chemo–resistant CRC spheroids and their counterparts and the precipitated DNA fragments were detected by PCR. Normal IgG was used as negative control. (H) Re–attached CRC cells were transfected with TOX3 siRNAs or overexpression plasmids or nonspecific control siRNAs or plasmids for 48 h, and the expression of ABCG2 and TOX3 was analyzed by western blot. The underlying data for Fig 1B, 1F and 1G can be found in S1 Data. The data is presented as the mean ± SD. *P < 0.05, **P < 0.01, ***P < 0.001, significant difference between treatment group and control group. AC, after chemotherapy; AM, after metastasis; BC, before chemotherapy; BM, before metastasis; cCSC, colorectal cancer stem cell; CRC, colorectal cancer; SD, standard deviation.

To further explore the molecular characteristics of these formed cCSCs, mRNA profile analysis was performed and compared between cCSCs and the corresponding adherent cells. As shown in Fig 1A, hierarchical cluster analysis showed consistent down- or up-regulated genes in cCSCs in both 2 cell lines from RNA-seq data (S1 and S2 Tables). The RNA-Seq analysis of tumor tissues from CRC patients sensitive and insensitive to chemotherapy also found some genes with higher or lower expression levels in patients with insensitive CRC to chemotherapeutics than those sensitive (S1F Fig). Of note, in both RNA-Seq analyses, ABCG2 commonly appeared among the up-regulated gene sets in the formed cCSCs and insensitive CRC tissue in response to stem-like traits accumulation and chemoresistance. Given its known function in mediating drug resistance, we speculated its critical role in the establishment and accumulation of CRC stem-like traits. Immunohistochemical results showed that ABCG2 had a higher expression level in the tissues of patients with recurrent or metastatic CRC (Fig 1B). Intriguingly, ABCG2 expression was gradually increased in serial passages of LOVO, HCT116, or RKO spheroids (Fig 1C). Collectively, in regard to the function of ABCG2 as one of the efflux transporter family, which has the ability to excrete cytotoxic drugs from cells, so as to be considered as a drug-resistant marker, and also a marker related to the inherent resistance or acquired resistance to chemotherapeutics in CSCs, we presume that ABCG2 might serve as a main contributor to promote colorectal cancer cell stem-like traits and drug resistance and plays a pivotal role in CRC development.

### TOX3 transcriptionally regulates ABCG2 in cCSCs

In attempt to explore the responsible regulatory mechanism for ABCG2 overexpression in the formed cCSCs, DNA Pull-Down assay was employed to fish the specific upstream regulatory factors of ABCG2 gene, which showed stronger binding capabilities at ABCG2 promoter (−861 to +360) in the formed cCSCs, compared to the attached cells. The obvious differential protein band with the molecular weight of 55 to 72 kd based on silver staining in Fig 1D was analyzed by mass spectrometry and proteomic techniques and identified to be TOX3, a transcriptional coactivator to be able to activate the transcription and expression of downstream target genes. The binding of TOX3 to the promoter region of ABCG2 in the formed cCSCs was confirmed (Fig 1E), and the precise binding site (−261 to −141 segments of the ABCG2 promoter region) was also identified (Figs 1F, 1G, S1G and S1H). Knockdown or overexpression of TOX3 down- or up-regulated ABCG2 level accordingly in the formed cCSCs (Fig 1H). In addition, a positive expression correlation between TOX3 and ABCG2 in CRC tissues was shown based on database analysis (S1I Fig).

### TOX3 facilitates colorectal cancer initiation and progression by up-regulating ABCG2

**Fig 2:**
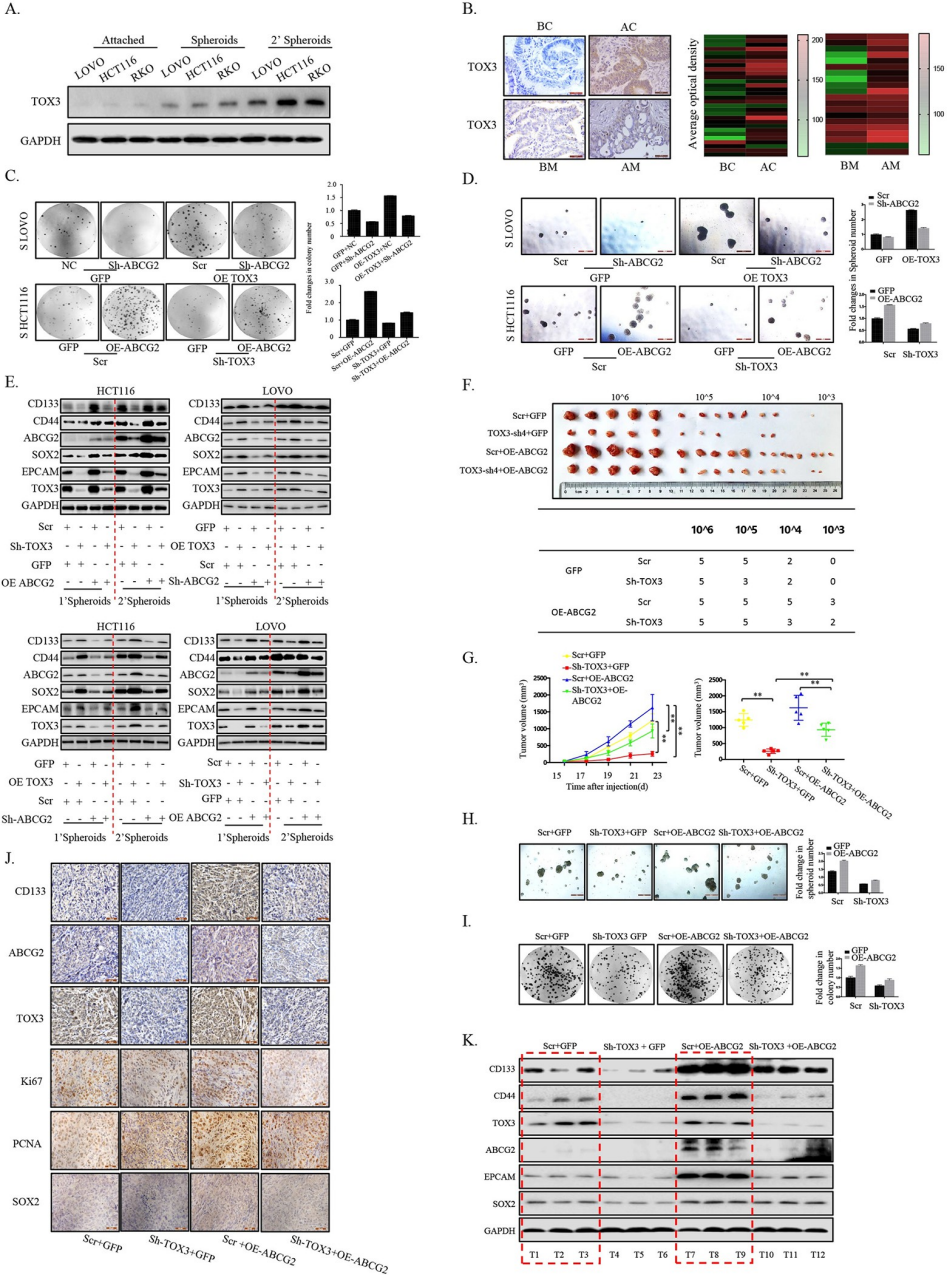
TOX3 promoted cCSCs expansion by up–regulating ABCG2. (A) Expressions of TOX3 in 2 successive generations of CRC spheroids and attached cells were examined by western blot. (B) The representative IHC staining of TOX3 and the scores of all the IHC staining in tumor tissues of CRC patients, including those BC, recurrent AC, primary tumor BM, and metastases AM. The heat map is a statistical map of the averaged optical density values of the slices stained with TOX3 antibody from 50 patients with or without chemotherapy (*n* = 30) and with or without metastasis (*n* = 20). (C, D) LOVO cells with stable overexpression of TOX3 and/or down–regulation of ABCG2 and HCT116 cells with stable down–regulation of TOX3 and/or overexpression of ABCG2 were subjected to colony and spheroid formation assay. The fold change of colony or sphere formation was quantified. (E) Bi–stable cell lines (HCT116 and LOVO) dissociated from the first and the second generation spheroids were subjected to western blot assay to test the expression of stem–like traits markers (CD133, CD44, SOX2, EPCAM) and ABCG2. (F) LDAs in a mouse model with xenografts of HCT116 cells with stable down–regulation of TOX3 and/or overexpression of ABCG2. cCSCs frequency was counted according to the number of the formed tumors. (G) Tumor diameters were measured at a regular interval of 2 days for up to 23 days, and the tumor volume was calculated. (H, I) CRC cells from xenografts of 4 different groups were digested, and the formation of colony and spheroid was evaluated 2 weeks post digestion. (J) Immunohistochemistry staining was used to detect the expression of CD133, ABCG2, TOX3, Ki67, PCNA, and SOX2 in the formed xenografts. (K) The expressions of stem–like traits markers (CD133, CD44, SOX2, EPCAM), TOX3 and ABCG2 were detected by immunoblot in the formed xenografts. The underlying data for 2C and 2D and 2G and 2H can be found in S1 Data. The data represent the mean ± SD of 3 independent experiments (*P < 0.05, **P < 0.01). AC, after chemotherapy; AM, after metastasis; BC, before chemotherapy; BM, before metastasis; cCSC, colorectal cancer stem cell; CRC, colorectal cancer; LDA, limited dilution assay; SD, standard deviation.

To explore the role of TOX3/ABCG2 signaling axis in CRC, we firstly investigated the function of TOX3 itself in cancer development. Analysis of TCGA and GEO databases showed that TOX3 was highly expressed in patients with CRC (S2A Fig), and its expression was up-regulated in the self-renewing spheroids compared with the attached cells and gradually increased in serial passages of CRC cell lines (Figs 2A and S2B). Furthermore, in line with the ABCG2 expression level, TOX3 showed a high expression in the recurrent or metastatic tumor tissues of patients with CRC (Fig 2B). Next, we found the clonogenicity, cell viability, and spheroid-forming ability of the formed cCSCs was decreased in CRC cells respectively with TOX3 knockdown (S2C–S2E Fig). By contrast, the overexpression of TOX3 in CRC spheroids showed increased clonogenic survival, cell viability, and spheroids formation than control cells (S2C–S2F Fig). Compared with the control, CRC spheroids derived from the cancer cells with TOX3 knockdown or overexpression showed decreased or elevated expression of ABCG2 and stem-like traits-associated protein markers (S2G Fig). Consistently, CRC cells with stable knockdown of TOX3 also showed the decreased clonogenic survival and spheroids formation and the down-regulated stem-like traits-related markers (S3A–S3E Fig), which further supports that TOX3 could facilitate the cCSCs expansion.

To further elucidate the key role of TOX3/ABCG2 signaling pathway in cCSCs expansion, we performed rescue assay in different CRC cell lines. Compared to control cells, TOX3 knockdown caused inhibited clonogenicity, lower spheroid formation, and the down-regulated level of the stem-like traits-associated factors, while ABCG2 overexpression rescued these trends. Accordingly, ABCG2 knockdown suppressed the clonogenicity, lowered spheroid formation, and down-regulated the expressions of the stem-like traits-associated factors, which were elevated by TOX3 overexpression whether in the first generation or the second generation (Figs 2C–2E and S3F). Both reversal systems further support that TOX3 promoted cCSCs expansion by targeting ABCG2.

The in vivo limited dilution assay (LDA) was also performed using CRC cells disassociated from cultured spheroids to further characterize the role of TOX3/ABCG2 signaling axis in CRC initiation and progression. In line with the in vitro results, TOX3 knockdown attenuated tumorigenicity and reduced the cCSCs frequency in comparison with the cells from control spheroids, which was reversed by ABCG2 overexpression, although ABCG2 overexpression led to the strongest tumorigenicity and stem-like traits (Fig 2F and 2G). Furthermore, the primary CRC cells were obtained from the formed xenograft tumor masses, and the same results were shown in the ability of spheroid formation and colony formation as in vitro upon TOX3 knockdown and/or ABCG2 overexpression (Figs 2H, 2I and S3G). Accordingly, in the formed xenografts, TOX3 knockdown down-regulated the level of the stem-like traits-associated and proliferation-associated factors, while ABCG2 overexpression reversed this trend (Fig 2J and 2K). Combined with the in vitro analysis, these data further indicate that TOX3/ABCG2 signaling axis promoted the expansion of cCSCs.

### TOX3/ABCG2 signaling axis expedites drug resistance in colorectal cancer

**Fig 3:**
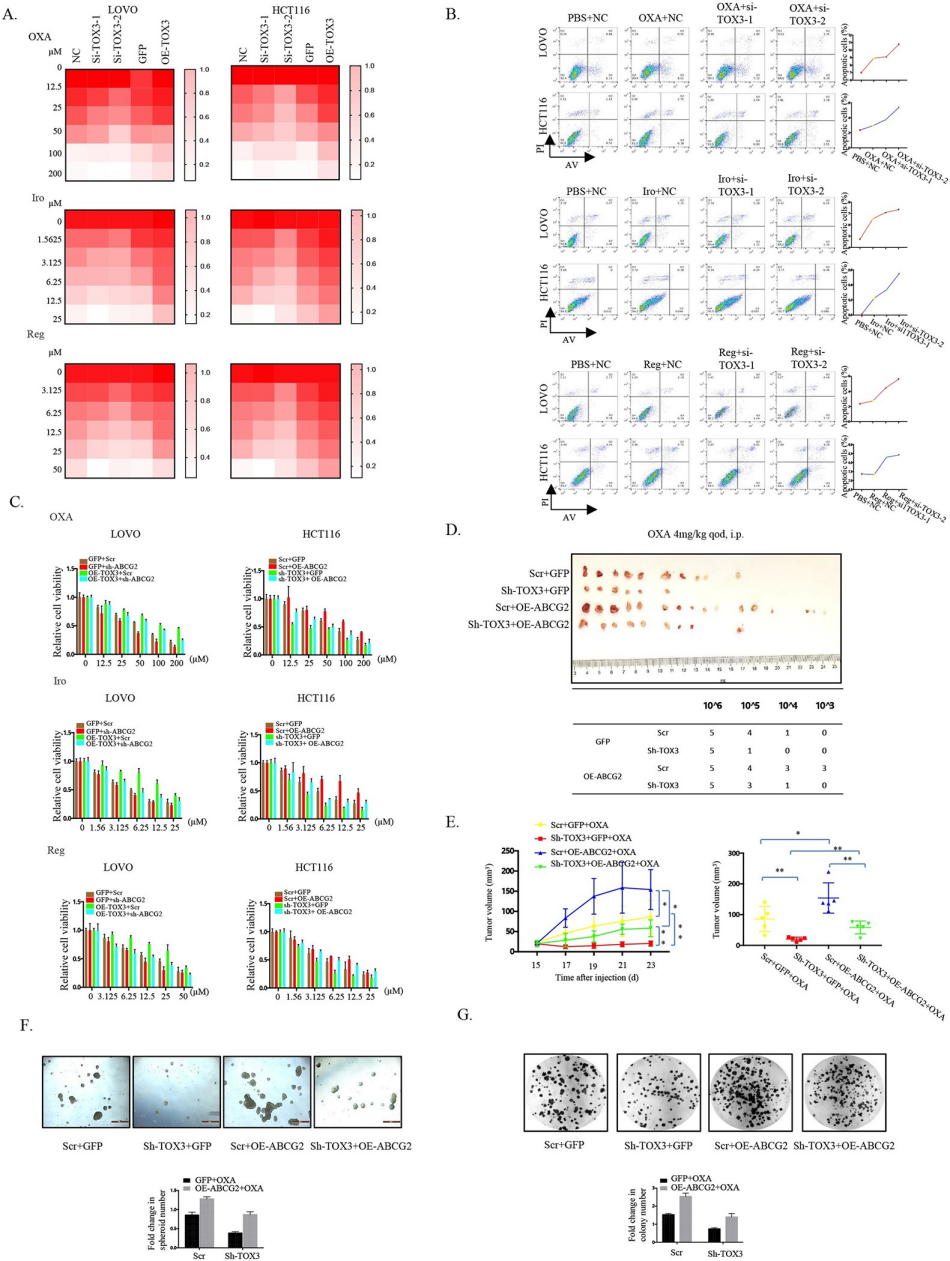
TOX3 knockdown sensitizes CRC cells to the treatment of chemotherapeutics and targeted agent by targeting ABCG2. (A) The sensitivity of LOVO and HCT116 cells to oxaliplatin, irinotecan, or regorafenib treatment at different concentrations was detected by MTT assay when TOX3 was silenced or overexpressed. (B) Flow cytometry analysis was used to evaluate cell apoptosis in LOVO and HCT116 cells with or without oxaliplatin, Irinotecan, or Regorafenib treatment when TOX3 was silenced or overexpressed, and the apoptotic cell number was calculated. (C) The cell viability assays were performed when HCT116 or LOVO cells with stable knockdown of TOX3 and overexpression of ABCG2 or stable overexpression of TOX3 and knockdown of ABCG2 were treated with different drugs (oxaliplatin, irinotecan, or regorafenib). (D) The morphology of tumor xenografts formed by HCT116 cells with stable knockdown of TOX3 and overexpression of ABCG2 upon treatment of oxaliplatin (4 mg/kg), and the LDA assay was also used to calculate cCSC formation frequency. (E) Tumor diameter of nude mice from each group was measured at a regular interval of 2 days for up to 23 days, and the tumor volume was calculated. (F, G) CRC cells from tumor xenografts of 4 different groups were digested, and the formation of spheroids and colony under oxaliplatin (1 μg/ml) treatment was evaluated 2 weeks post dissociation. The underlying data for Fig 3A, 3C and 3E–3G can be found in S1 Data. The data is presented as the mean ± SD. *P < 0.05, **P < 0.01, ***P < 0.001, significant difference between treatment group and control group. cCSC, colorectal cancer stem cell; CRC, colorectal cancer; LDA, limited dilution assay; SD, standard deviation.

Given that as a drug-resistant molecule, ABCG2 is involved in single-drug and multidrug resistance in different types of tumors, we supposed that TOX3 similarly promotes drug resistance in CRC based on its regulation on ABCG2 and the contribution of TOX3/ABCG2 signaling axis in maintaining cancer cell stem-like traits. When challenged with gradient concentration of clinical CRC drugs, including chemotherapeutics, oxaliplatin and irinotecan and targeted agent, regorafenib, CRC cells with TOX3 knockdown exhibited enhanced drug sensitivity, whereas TOX3 overexpression suppressed the sensitivity to these clinical drugs (Figs 3A and S4A). Moreover, we also found that the sensitivity of CRC cells to oxaliplatin was time-dependent upon TOX3 knockdown or overexpression (S4B Fig). Compared to control cells, TOX3 knockdown not only increased the inactivation of clonogenicity mediated by oxaliplatin treatment, but also boosted the cCSCs expansion suppression triggered by oxaliplatin treatment (S4C and S4D Fig). In addition, more apoptosis was induced in CRC cells with TOX3 knockdown when exposed to different drugs (Fig 3B), implying that TOX3 expression was indeed involved in drug resistance evolution in CRC cells.

To further confirm the role of TOX3/ABCG2 signaling axis in drug resistance development during CRC treatment, rescue experiments were employed again. When exposed to drugs, TOX3 knockdown significantly improved the impaired cell growth, clonogenicity, and spheroid formation caused by agent treatment, whereas the overexpression of ABCG2 reversed such effect. Consistently, TOX3 overexpression recovered the decreased cell growth, clonogenicity, and spheroid formation upon drug treatment, which also could be reversed by knockdown of ABCG2 (Figs 3C and S5A–S5F). Furthermore, LDA in mice also indicated that TOX3 knockdown improved tumor growth inhibition caused by oxaliplatin, while ABCG2 overexpression abolished such decrease in cCSCs expansion triggered by TOX3 knockdown upon oxaliplatin treatment compared with the control group (Fig 3D and 3E), and the same results were obtained when primary cells were digested from xenografts and used for spheroid formation assay, clonogenicity, and CSC frequency analyses (Figs 3F, 3G and S4E). Taken together, these data demonstrated that TOX3 functions as a critical upstream regulatory factor of ABCG2 to facilitate the drug resistance of CRC cells.

### TOX3 recruits WDR5 to co-anchor at ABCG2 promoter region through tri-methylation of H3K4 in cCSCs

**Fig 4:**
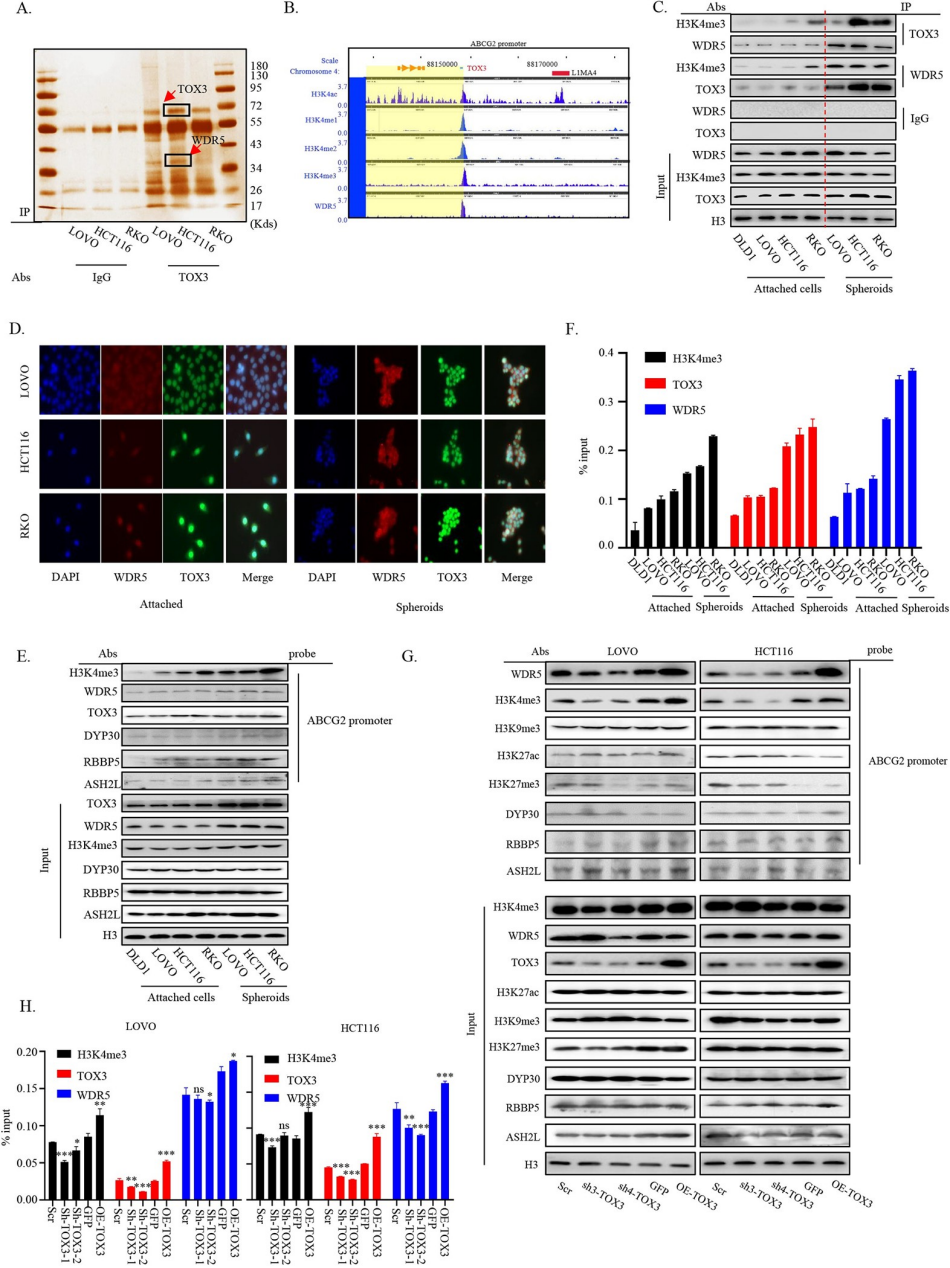
TOX3 promotes WDR5 recruitment at the ABCG2 promoter in cCSCs through tri–methylation of H3K4. (A) The silver staining showed the binding proteins of TOX3 after IP assay in the cCSCs. The arrow indicates the candidate binding protein analyzed by MS. (B) WashU Epigenome Browser tracks of the 5 proteins and their binding peaks within the examined ABCG2 promoter region in CRC cells. (C) The interaction in pairs among TOX3, WDR5, and H3K4me3 was detected by IP assay in the cCSCs and their counterparts (attached cells cultured without oxaliplatin). Normal IgG was used as a negative control. (D) Co–localization of TOX3 and WDR5 in attached or spheres of CRC cells by immunofluorescence assay. (E) Pull–down assay was performed to detect the binding of TOX3, WDR5, H3K4me3, RBBP5, ASH2L, and DPY30 to ABCG2 promoter in the chemo–resistant CRC spheroids and their counterparts. (F) ChIP assay was performed to detect the binding of TOX3, WDR5, and H3K4me3 to ABCG2 promoter in the chemo–resistant CRC spheroids and their counterparts. (G) Pull–down was performed to detect the binding of TOX3, WDR5, H3K4me3, RBBP5, ASH2L, DPY30, H3K9me3, H3K27ac, and H3K27me3 to ABCG2 promoter in the reattached CRC cells (LOVO and HCT116 cell lines) from spheroids with down–or up–regulation of TOX3. (H) ChIP assay was performed to detect the binding of TOX3, WDR5, and H3K4me3 to ABCG2 promoter in the reattached CRC cells (LOVO and HCT116 cell lines) from spheroids with down–or up–regulation of TOX3. The underlying data for Fig 4F and 4H can be found in S1 Data. The data is presented as the mean ± SD. *P < 0.05, **P < 0.01, ***P < 0.001, significant difference between treatment group and control group. cCSC, colorectal cancer stem cell; CRC, colorectal cancer; SD, standard deviation; WDR5, WD Repeat Domain 5.

To further clarify the potential regulatory mechanism of TOX3 on ABCG2 in the initiation and development of cCSCs, IP-MS was used to explore the interacting protein of TOX3 in the spheroid cultures of CRC cells. As shown in Fig 4A, a 35 kd protein, WD Repeat Domain 5 (WDR5), identified by MS, was focused, based on its known function in promoting the methylation of Lys4 on histone 3 (H3K4) and inducing the expression of oncogenes. WDR5 was found to be highly expressed in CRC according to the analysis from GEO and TCGA databases (S6A Fig), and its high expression was positively correlated with the poor prognosis of CRC patients (S6B Fig). A weak expression correlation between WDR5 and TOX3 was shown based on the correlation analysis from the database (S6C Fig). However, in agreement with TOX3, WDR5 showed comparatively high expression in patients with metastatic or relapsed CRC (S6D Fig). Interestingly and worth mentioning that, according to ChIP-seq database, both H3K4me3 and WDR5 were found to bind to the ABCG2 promoter region in CRC cells, and the binding position just includes the segments of −261 to −141, which was similarly bound by TOX3 within ABCG2 promoter (Fig 4B). We therefore hypothesized that TOX3 cooperates with WDR5 to methylate H3K4 at ABCG2 promoter, resulting in the up-regulation of ABCG2 transcription, cCSCs expansion, and ultimately in the drug resistance development. IP assay confirmed the interaction between WDR5/H3K4me3 and TOX3, and of note, such interactions were increased when the CRC spheroids were formed (Fig 4C). Consistently, the co-localization signals were shown between WDR5 and TOX3 in different CRC cell cultures and much stronger expression and co-localization signals were seen in spheroids, compared to the attached cells (Fig 4D). We next determined whether TOX3 cooperated with WDR5/H3K4me3 to promote ABCG2 transcription. As expected, not only WDR5 and H3K4me3, but also the other members of the WRAD complex, could bind to the same site of ABCG2 promoter as TOX3, and the binding showed a stronger trend in the formed cCSCs (Fig 4E and 4F). Moreover, we observed that TOX3 knockdown attenuated the binding capacity of WDR5 and H3K4me3 at ABCG2 promoter, whereas its overexpression increased such binding levels in CRC cells (Figs 4G, 4H, S6E and S6F). By contrast, the binding of the other members of the WRAD complex and some other epigenetic markers at ABCG2 promoter was not affected by TOX3 expression change (Fig 4G).

Meanwhile, the bindings of TOX3 and H3K4me3 at ABCG2 promoter were suppressed when WDR5 was silenced, and their bindings were increased upon WDR5 overexpression. Of note, the suppressed bindings were observed when WDR5 inhibitor, OICR-9429 was used (S6G and S6H Fig), suggesting that tri-methylation of H3K4 induced by WDR5 promotes the binding of TOX3 at ABCG2 promoter, either directly or indirectly. These results collectively demonstrated the synergy of WDR5 in the anchoring of TOX3 at ABCG2 promoter through methylating H3K4.

### The regulation of TOX3 on ABCG2-induced stem-like traits and drug resistance depends on the tri-methylation of H3K4 mediated by WDR5

Given the results above, we assumed that WDR5 tri-methylates H3K4 at ABCG2 promoter to further recruit TOX3 resulting in the up-regulation of ABCG2 transcription and ultimately in the development of cancer stem-like traits and drug resistance. Thus, we next determined the role of WDR5 itself in mediating ABCG2 expression and subsequent cCSCs expansion and drug resistance. We found cells with WDR5 knockdown showed the lower transcription activity of ABCG2, the inhibited spheroid formation, clonogenicity, the reduced CD133, CD44, SOX2, EPCAM, and ABCG2 expression, but nearly unchanged level of TOX3, and the enhanced sensitivity to oxaliplatin (S7A–S7F Fig). By contrast, the transcription activity of ABCG2, spheroid formation, clonogenicity, and the expression of cancer stem-like traits-related genes was respectively enhanced, and the sensitivity to oxaliplatin was attenuated, in CRC cells by WDR5 overexpression. More significantly, the same change trend was seen upon OICR9429 treatment, a high affinity and selective WDR5 antagonist, as WDR5 knockdown, in CRC cells (S7A–S7F Fig). These data showed the contribution of WDR5 itself in promoting ABCG2 expression and subsequent cCSCs expansion and drug resistance via mediating the tri-methylation of H3K4.

**Fig 5:**
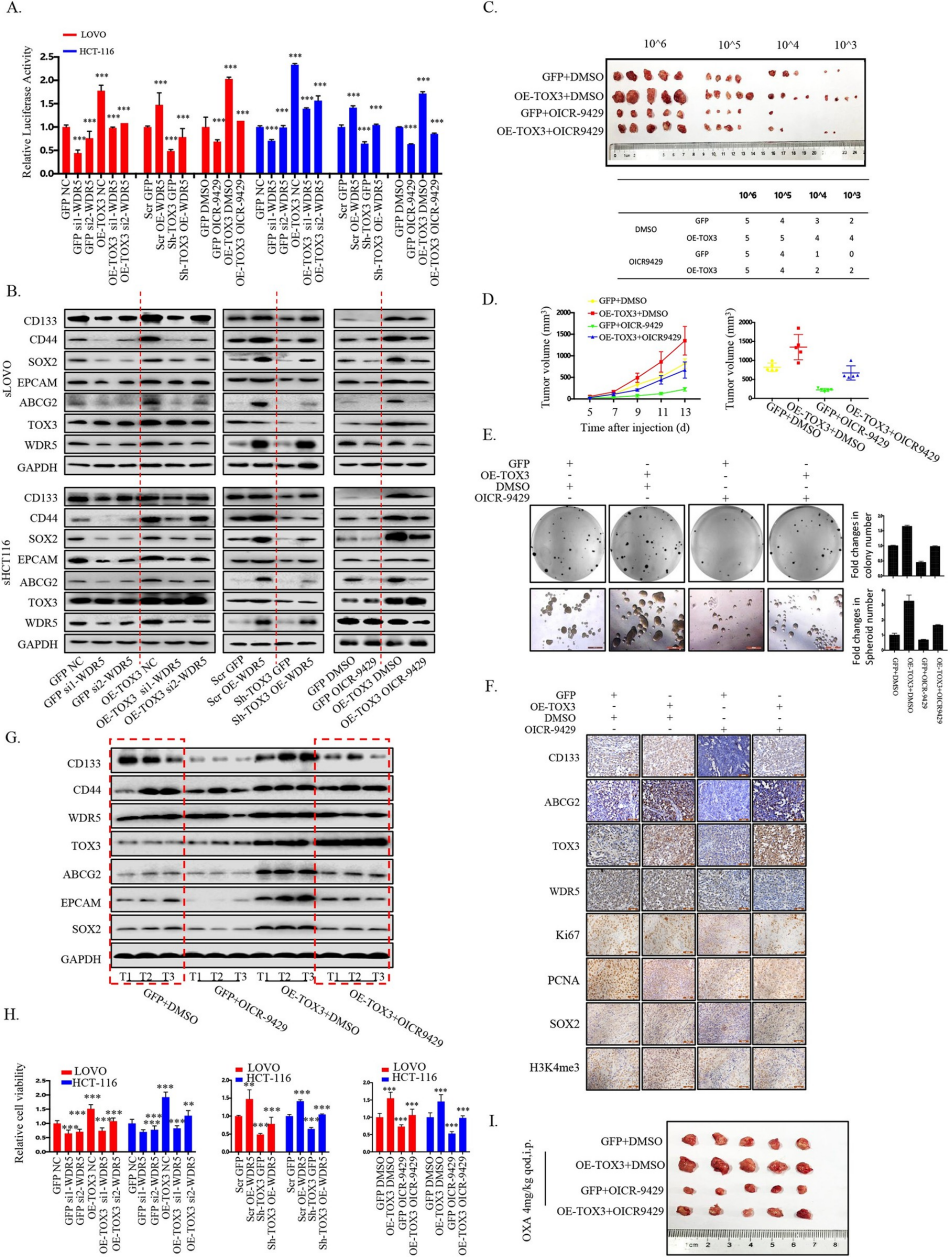
TOX3 synergized with WDR5 to co–promote cCSCs expansion and drug resistance in vivo and in vitro. CRC cell lines with stable knockdown or overexpression of TOX3 were treated with WDR5–overexpressing plasmids or si–WDR5 or OICR–9429, and then (A) ABCG2 promoter–driven luciferase reporter assay was performed. (B) Immunoblot assay was used to detect the expression of stem–like traits markers (CD133, CD44, SOX2, EPCAM, ABCG2, WDR5, and TOX3) in re–attached CRC cells. (C) LOVO cells with stable overexpression of TOX3 or not were used to perform LDAs, and mice were treated with OICR9429 (1.5 mg/kg per day) for 7 consecutive days. The cCSCs frequency was counted finally. (D) Tumor diameters were measured at a regular interval of 2 days for up to 13 days, and the tumor volume was calculated. (E) CRC cells from xenografts of 4 different groups were digested, and the formation of colony and spheroids was evaluated 2 weeks post digestion. (F) Immunohistochemistry staining was used to detect the expression of CD133, ABCG2, WDR5, TOX3, Ki67, PCNA, SOX2, and H3K4me3 in the formed xenografts. (G) The expressions of stem–like traits markers (CD133, CD44, SOX2, EPCAM), ABCG2, WDR5, and TOX3 were detected by immunoblot assay in xenografts. (H) The sensitivity of CRC cells with stable overexpression of TOX3 and knockdown or inactivity of WDR5 or with stable knockdown of TOX3 and overexpression of WDR5 to oxaliplatin treatment was detected by MTT assay in CRC cell lines. (I) The morphology of tumor xenografts formed by LOVO cells with stable TOX3 overexpression or not under treatment of OICR–9429 processed as described before. Meanwhile, the mice were treated by oxaliplatin (4 mg/kg) for 2 weeks. The underlying data for Fig 5A, 5D–E, and 5H can be found in S1 Data. The data is presented as the mean ± SD. *P < 0.05, **P < 0.01, ***P < 0.001, significant difference between treatment group and control group. cCSC, colorectal cancer stem cell; CRC, colorectal cancer; LDA, limited dilution assay; SD, standard deviation; WDR5, WD Repeat Domain 5.

Next, rescue assay was performed to further verify our assumption. TOX3 overexpression reversed the inhibition at the transcription of ABCG2, the expression of cancer stem-like traits-related genes, clonogenicity, spheroid formation, and CSC frequency induced by WDR5 knockdown, and vice versa (Figs 5A, 5B and S8A–S8D). It is worth noting that, in line with WDR5 knockdown, WDR5 inactivity effectively reversed the enhancement of the promoter activity of ABCG2, the stem-like traits-related gene expressions, clonogenicity, and spheroid formation induced by TOX3 overexpression (Figs 5A, 5B, S8E and S8F). Furthermore, the synergy between TOX3 and WDR5/H3K4me3 in maintaining ABCG2 expression and stem-like traits of CRC cells was evaluated in nude mice model. LDA assay showed TOX3 overexpression increased stem-like traits of the tumor cells and promoted tumor growth, but OICR9429 treatment reversed this promotion (Fig 5C–5E). In addition, immunohistochemistry and western blot assay revealed the increased levels of ABCG2, CD133, and other stem-like traits-related and proliferation-related markers resulted by TOX3 overexpression were also decreased by OICR-9429 treatment (Fig 5F and 5G).

Then, we explored the effect of TOX3/WDR5 signaling axis on the efficacy of oxaliplatin on CRC cells. Our results showed that WDR5 knockdown or inactivity reversed the down-regulated sensitivity of CRC cells to oxaliplatin caused by TOX3 overexpression, while WDR5 overexpression desensitized cancer cells to oxaliplatin based on TOX3 knockdown (Fig 5H). The same results were shown in tumor-bearing mice with oxaliplatin treatment (Figs 5I, S8G, and S8H). Collectively, our results demonstrate that TOX3 synergized with WDR5 to co-regulate ABCG2 transcription and subsequent stem-like traits maintenance and drug resistance for CRC and such regulation was dependent on the tri-methylation of H3K4 mediated by WDR5.

### TOX3-WDR5/ABCG2 signaling pathway is abnormally activated during oxaliplatin resistance production in AOM/DSS-induced in situ colorectal cancer mouse model

**Fig 6:**
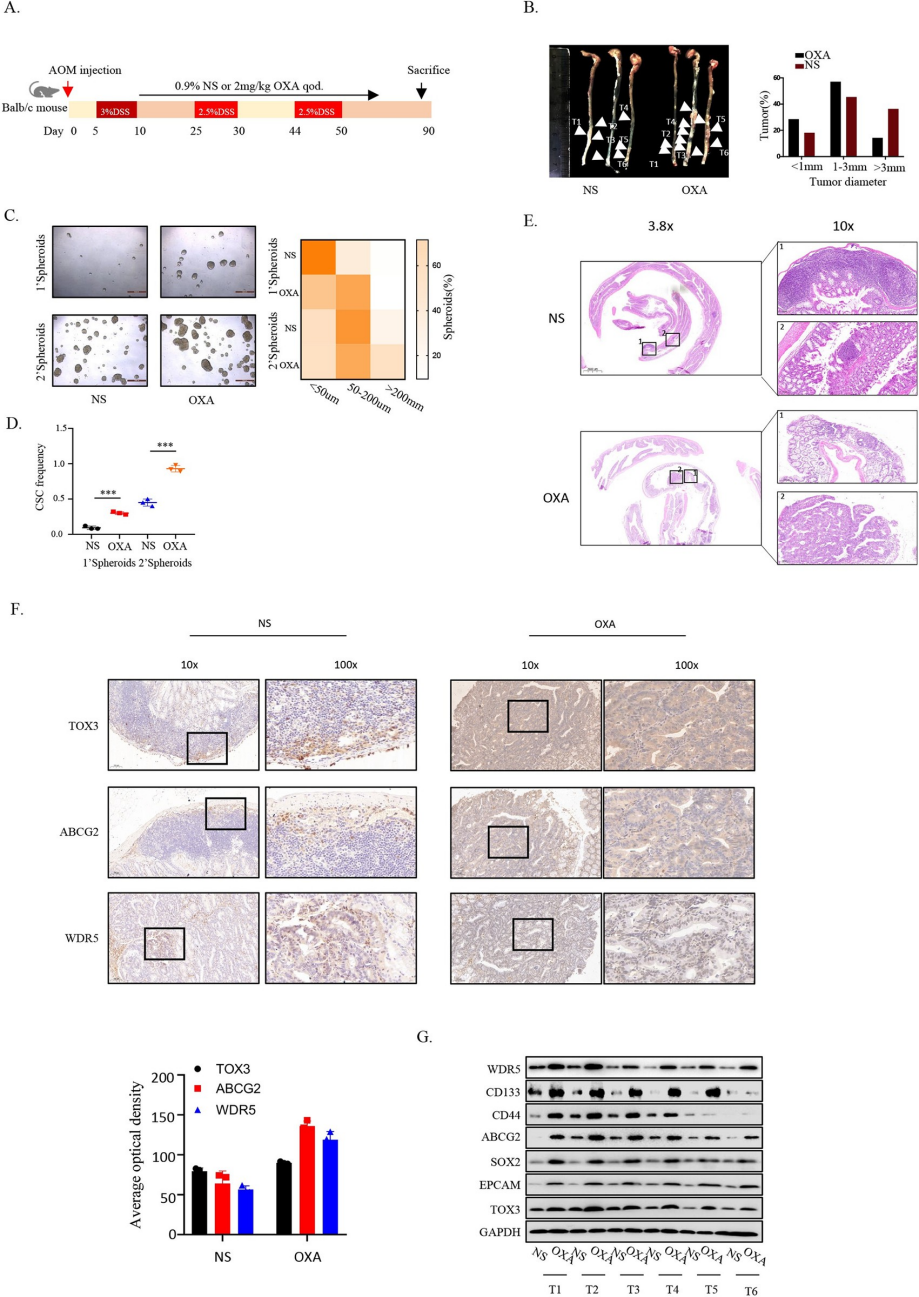
Oxaliplatin–resistant CRC in situ tumor shows high expression of ABCG2, WDR5, and TOX3. (A) Schematic illustration of the experimental approach applied to assess the tumorigenicity in Balb/c mice induced by AOM/DSS. (B) Intestines of mice with tumor were shown in NS and OXA treatment group 90 days after AOM injection. The number and size of the formed tumors were calculated respectively (*n* = 3). (C). CRC cells were digested from the formed tumors and spheroid culture was performed for 2 passages. The spheres were imaged and quantitatively analyzed after 4 weeks. (D) The frequency of cCSCs was calculated by in vivo limiting dilution assay in which cells were digested from xenograft. (E) HE staining of the entire intestine of mouse by Swiss–rolling technique. (F) Immunohistochemical staining was used to detect the expression of TOX3, ABCG2, and WDR5 in the formed intestinal tumors, and averaged optical density was calculated. (G) Immunoblot assay was used to detect the expression of stem–like traits markers (CD133, CD44, SOX2, EPCAM), ABCG2 and TOX3 in the formed intestinal tumors of mice with different treatment. The underlying data for Fig 6B–6D and 6F can be found in S1 Data. cCSC, colorectal cancer stem cell; CRC, colorectal cancer; WDR5, WD Repeat Domain 5.

To further investigate the role of TOX3-WDR5/ABCG2 signaling pathway in CRC stem-like traits maintenance and drug resistance development, we established a mouse model of colitis-associated colorectal tumorigenesis, in which mice were injected intraperitoneally with AOM, followed by 3 rounds of DSS treatment, and were treated with 0.9% NS or 2 mg/kg OXA qod, followed by first DSS and the development of colon tumors were examined after 80 days (Fig 6A). Compared to NS treatment mice, OXA treatment mice developed tumors with significantly increased numbers and sizes throughout the middle and distal colon (Fig 6B). Considering the reported theory that CRC originates from intestinal stem cells and the tight correlation between stemness and chemotherapeutic resistance [32–35], we hypothesized that the dramatic increase in cell proliferation and tumor formation in OXA treatment mice was caused by a population of intestinal stem cells that is unsusceptible to OXA. The in vitro tumor spheroid culture indicated that cells harvested from dysplasias of OXA treatment mice showed sustained and much stronger spheroid-forming capacity compared with those harvested from NS treatment mice (Fig 6C and 6D). In addition, based on HE staining, low-grade dysplasia was restricted to tumor-bearing mice with NS treatment, whereas both low- and high-grade dysplasia was observed in OXA treatment mice (Fig 6E). Consistently, the expressions of genes related to cancer stem-like traits were increased in the colorectal tumors of OXA treatment mice, compared to NS treatment mice, including TOX3, WDR5, and ABCG2 (Fig 6F and 6G). Altogether, these data indicated that the in situ CRC with OXA resistance exhibited a serial of increased stem-like traits accompanied by the abnormal activation of TOX3-WDR5/ABCG2 signaling pathway.

### Both TOX3 and ABCG2 up-regulation predicts poor prognosis of CRC patients

**Fig 7:**
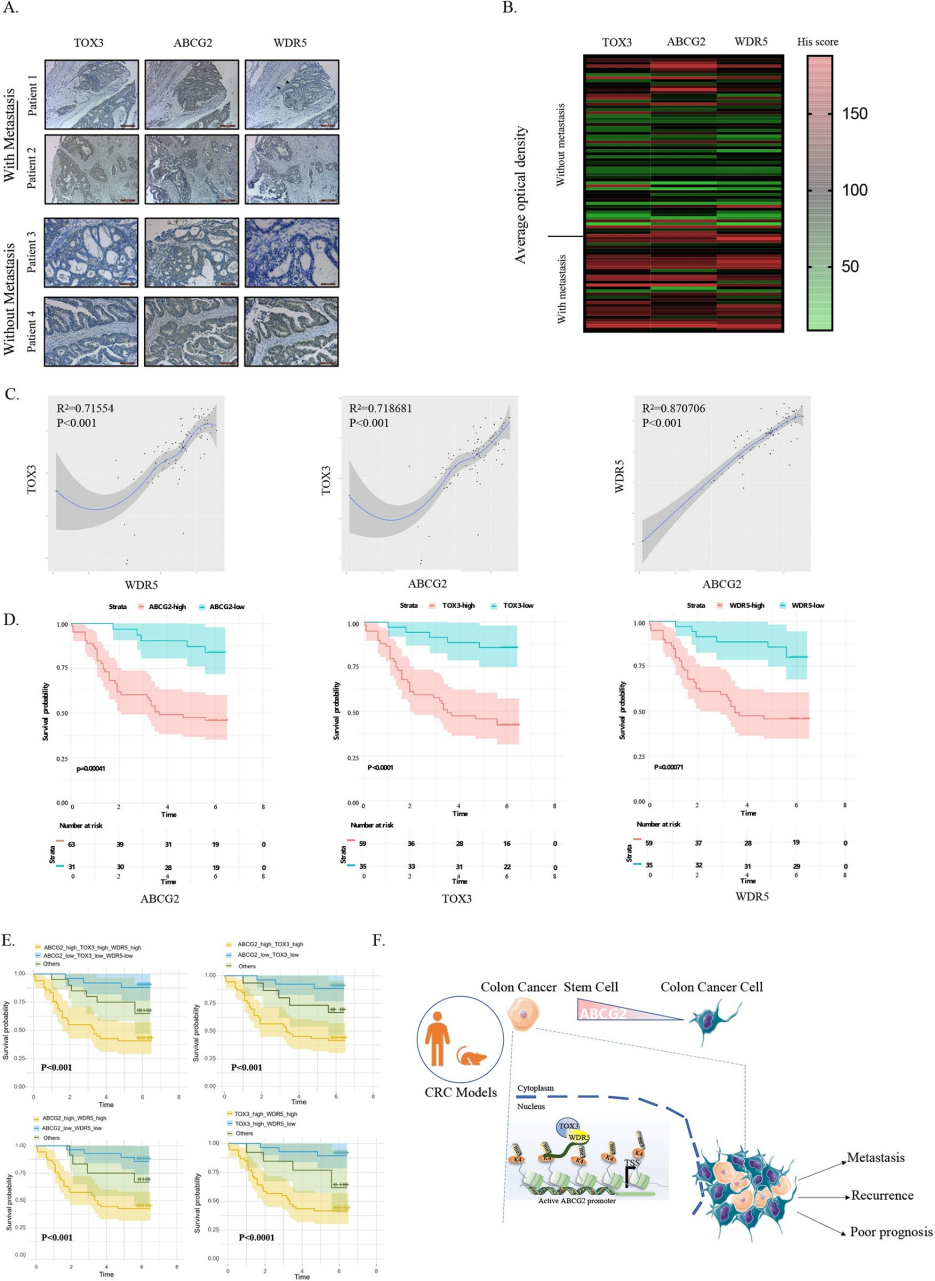
The high expression of ABCG2 was positively correlated with TOX3 and WDR5 expression in CRC tissues and predicted poor prognosis in patients with CRC. (A, B) The immunohistochemical staining analysis of TOX3, WDR5, and ABCG2 protein expression from human CRC tissue microarray. The representative images and averaged optical density for high and low expression were shown. (C) The correlation among the expression of TOX3, WDR5, and ABCG2 in human CRC tissues based on IHC attaining of tissue microarray. (D) Kaplan–Meier analysis showing the overall survival of CRC patients harboring high (red) or low (blue) TOX3, WDR5, or ABCG2 expression (*P* < 0.001, log–rank test). (E) Kaplan–Meier analysis showing the overall survival of CRC patients harboring simultaneous high or low TOX3, WDR5, and ABCG2 expression or harboring simultaneous high or low expression of any 2 among these 3 proteins (*P* < 0.001, log–rank test). (F) The schematic diagram illustrating the related molecular mechanisms of TOX3–WDR5–H3K4me3/ABCG2 signaling axis involved in cCSCs expansion, CRC recurrence, and metastasis. The underlying data for Fig 7B–7E can be found in S1 Data. cCSC, colorectal cancer stem cell; CRC, colorectal cancer; WDR5, WD Repeat Domain 5.

To clarify the clinical significance of TOX3-WDR5/ABCG2 signaling axis in CRC progression, we divided the 94 patients into 2 groups based on the CRC tissue microarray, with or without lymph node metastasis for clinical analysis. As shown in Fig 7A and 7B, the increased TOX3, WDR5, and ABCG2 expression was respectively detected in the majority of metastatic CRCs as compared with those with no lymph node metastasis. Pearson correlation analysis suggested the potential positive expression correlation between any 2 of these 3 proteins, TOX3, ABCG2, and WDR5, in this cohort of 94 patients (Fig 7C). Furthermore, the univariate analysis demonstrated that the patients with higher TOX3, ABCG2, or WDR5 level possessed a higher risk of CRC recurrence and a shorter survival time after surgical resection (Fig 7D and S3–S5 Tables). Furthermore, the simultaneous high or low expression of TOX3, ABCG2, and WDR5 or the simultaneous high or low expression of any 2 among these 3 proteins was significantly correlated with poor prognosis of CRC patients (Fig 7E). More than that, we have also searched the clinicopathological data of 2,989 cases with CRC from The First Affiliated Hospital of Dalian Medical University and chose 50 patients with recurrence or metastasis to analyze the expression correlation between any 2 of these 3 proteins, TOX3, ABCG2, and WDR5, and clinicopathologic variables (S9 Fig and S6–S9 Tables) and disease-free survival rate (S9 Fig and S8 Table). The results showed the combination of TOX3, ABCG2, and WDR5 could be a more efficient predictor for the survival of CRC patients with recurrence or metastasis.

## Discussion

As a highly prevalent digestive system cancer, CRC shows a low survival rate at advanced stages of the disease. In recent years, CSCs have been found to play critical effects on tumor formation, heterogeneity, therapeutic resistance, and eventual recurrence and metastasis. However, our knowledge about the target genes involved in cCSCs expansion and therapeutic resistance and the related regulatory mechanisms for these genes remains largely limited. Other than cell sorting using specific cell surface markers, cCSCs could also be enriched according to their particular characteristics such as side population, low proteasome activity and low intracellular ROS, spheroid formation, or chemoresistance [36–38]. In the current study, cCSCs were enriched by spheroid formation upon chemotherapeutic treatment, and ABCG2 was focused among the up-regulated genes in cCSCs by comparation to the adherent cancer cells. Moreover, ABCG2 was further identified based on its elevated expression in chemo-insensitive, recurrent, and metastasized CRC tissues. In the subsequent study, our data showed that ABCG2 promoted the self-renewal and expansion of cCSCs, thereby contributing to drug resistance production and CRC progression.

It is well established that ABCG2 is a star marker of drug tolerance, and its overactivation has been found to occur in almost all tumors with drug resistance or metastasis, including CRC [39]. Nevertheless, as one of the most studied molecules, our understanding about the regulatory and signaling network of ABCG2 remains incomplete in CRC, especially regarding to its progressive role in cCSCs expansion and drug resistance. Here, the upstream regulatory mechanisms of ABCG2 in cCSCs were explored. Through DNA pull-down and MS analysis, multiple transcriptional factors were found to bind to the ABCG2 promoter, among which, TOX3 was selected and investigated. As one of the least well-characterized transcriptors, our knowledge about its function in cancer development is very limited. TOX3 is previously found to be a novel candidate regulator yielding specific enteroendocrine phenotypes [30]. In addition, it is also reported to improve the discriminatory accuracy of BCRAT modestly for breast cancer [40]. However, hitherto, the published work shows contradictory results relating to its impact on tumorigenesis, suggesting that more careful dissections are required for TOX3 in different cancer types and different stages of the same cancer type [28,41–43]. Our present results reveal that TOX3 is particularly abundant in cCSCs and metastatic and recurrent tumor tissues from CRC patients and is essential for cCSCs evolution and drug resistance development by transcriptionally up-regulating ABCG2 via binding at ABCG2 promoter, which suggests that TOX3/ABCG2 signaling may represent a new layer of control for cancer initiation and progression, particularly for CRC, by reshaping cancer cell stem-like traits and drug resistance.

Considering the function of TOX3 in regulating downstream target gene transcription by acting as a transcriptional coactivator and its reported interaction with the p300/CBP transcriptional complex [26], we reasonably supposed the existence of some synergizers in its regulation on ABCG2 transcription in cCSCs. Based on IP-MS analysis, WDR5 was found to interact with TOX3 and synergistically regulate the transcription and subsequent expression of ABCG2, thus promoting cCSCs expansion and drug resistance. Intriguingly enough, WDR5 not only showed the binding at the promoter of ABCG2 gene, but also showed the same binding site with TOX3 within ABCG2 promoter, implying the recruitment and necessity of WDR5 in the regulation of ABCG2 transcription mediated by TOX3. Furthermore, given the essential role of WDR5 in mediating the tri-methylation of H3K4 and transcriptional activation of target genes involved in pluripotency, development and cancer, and H3K4me3 functioning as a key histone modification that often selectively localizes to target gene promoters to activate target gene transcription [44,45], we explored and confirmed the dependence of TOX3 on the methyltransferase activity of WDR5 and tri-methylation of H3K4 mediated by it during the activation of ABCG2 transcription. As evidenced by pull-down and ChIP assays, the application of OICR-9429, a high affinity small molecule compound that can competitively block the interaction of WDR5 with MLL protein via binding the central peptide-binding pocket of WDR5, simultaneously reduced the enrichment of histone H3K4me3, WDR5, and TOX3 in the ABCG2 promoter region, leading to the inhibition of ABCG2 transcription. Thus, to our knowledge, it should be the first time to be fully characterized that TOX3/WDR5/H3K4me3 complex binds to ABCG2 promoter to co-control its transcription via chromatin modifications. In line with the established points that changes in the global genome-wide landscape of histone methylation are essential for cancer cell proliferation and stem-like traits during cancer onset and progression, we demonstrated the utility of TOX3/WDR5/H3K4me3 signaling axis as selective chromatin binders in CRC cells to promote cCSCs expansion and drug resistance by stably expressing ABCG2. Of note, in agreement, besides WDR5, the other members of the WRAD complex, such as ASH2L [ash2 (absent, small, or homeotic) like], RBBP5 (retinoblastoma-binding protein 5), and DPY30 (*C*. *elegans* dpy-30 homolog) [46] were also enriched at ABCG2 promoter, especially in cCSCs, but not in adherent cells (Fig 4E). However, unlike WDR5, their binding at ABCG2 promoter was not affected by TOX3 expression level (Fig 4G). Considering that although it has been reported that depletion of any component of the WRAD complex results in the global loss of H3K4 methylation to varying degrees [47–49], WRAD complex tends to be complicated with the evolution of species, and there might be other variable factors in different complexes [50]. Based on our current results, we could only preliminarily conclude that TOX3 was involved in the establishment and maintenance of the stem-like traits of CRC cells mainly by facilitating the transcription of ABCG2 via recruiting WDR5 and its subsequent promotion on the tri-methylation of H3K4 at ABCG2 promoter region. As for the engagement and contribution of the other component within the WRAD core catalytic complex during this transcriptional regulation and even during the stemness initiation and maintenance of CRC cells, all these issues deserve to be further explored later.

WDR5 has been well established to serve as an oncogene to be involved in tumorigenesis and development [51]. Thus, inhibitors targeting WDR5, especially those blocking its methyltransferase activity, present the possibility of being developed as anticancer agents. As one of these compounds, OICR-9429 showed anticancer efficacy by inhibiting cell vitality in colon and prostate cancer [52–54]. However, the biological functions affected by OICR-9429 in CRC stem-like traits maintenance remain unknown. In this study, we firstly discovered that OICR-9429 inhibited the stem-like traits of CRC cells by blocking ABCG2 activation induced by TOX3\WDR5\H3K4me3 complex, being manifested by the suppressed proliferative potential, the down-regulated stem-like traits-related protein markers and the attenuated spheroid-forming ability in cCSCs upon OICR-9429 treatment. Furthermore, consistent with the previous studies showing that epigenetic modification-associated proteins, such as HDAC6, EZH2, and H3K4me3, played a vital role in chemoresistance regulation, we also found that OICR-9429 suppressed tumor growth and promoted the chemosensitivity of oxaplatin in CRC cells in vivo, suggesting that the combined treatment of OICR-9429 and oxaliplatin could improve the curative effect and reduce the side effects of original chemotherapy. Therefore, to expand upon this, WDR5 depletion, combined with chemotherapeutics, could be considered as a promising candidate strategy in anti-CRC therapy to improve clinical outcomes by disrupting stem-like traits of cancer cells and amplifying the toxic effects of chemotherapeutics.

Since TOX3-WDR5/ABCG2 signaling axis has been found to play a vital role in cCSCs expansion and drug resistance according to our in vitro and in vivo research, and cancer stem-like traits serve as a molecular phenotype, which is closely related to tumor metastasis, spread and the poor clinicopathological characteristics, further exploring the clinical implications of this signaling axis seems necessary. Here, we reviewed the medical records of 2,989 patients with CRC, and finally screened 50 patients with drug resistance or metastasis, including 30 patients with metastasis and 20 patients with drug resistance after chemotherapy. Compared with normal tissues, whether ABCG2, TOX3, or WDR5 is up-regulated in clinical metastatic and drug-resistant tumor specimens, and shows a strong prognostic value for OS and DFS in drug-resistant CRC patients. Beyond this, the high expression level of ABCG2, TOX3, and WDR5 in CRC tissues and their prediction for patient survival was further confirmed based on the data from both the GEO dataset and the TCGA database. Combined with the results from the AOM/DSS-induced in situ CRC mouse model that the expression of ABCG2, TOX3, and WDR5 was elevated in oxaliplatin-resistant tumor tissues, our study provides a robust evidence support to develop TOX3-WDR5/ABCG2 signaling axis as the candidate biomarkers to predict the chemosensitivity and even the clinical outcomes of CRC patients.

To sum up, our results, for the first time, demonstrate the contribution of TOX3-WDR5-H3K4me3/ABCG2 signaling axis to CRC initiation and development by accelerating stem-like traits and drug resistance evolution (Fig 7F). The synergistic anchoring of TOX3 with WDR5 at the promoter of ABCG2 causes the enrichment of histone H3K4 tri-methylation, thereby further promoting ABCG2 transcription and expression and subsequent cancer stem-like traits expansion and drug sensitivity deregulation. Consistent with ABCG2, TOX3, or WDR5 itself also shows the abnormally high expression in the metastasized or recurrent CRC tissues after receiving chemotherapy, and their respective activation confers stem-like traits and drug insensitivity enhancement in CRC cells. The combination of TOX3-WDR5/ABCG2 signaling axis could not only be considered as a group of combinational candidate therapeutic targets in anti-CRC therapy, but also serve as a group of more efficient cascade predictors for the survival of CRC patients with recurrence or metastasis. Of course, with further study in the future, more aspects of the function for this signaling axis should be disclosed.

## Conclusions

In brief, our study uncovers that ABCG2 is overactivated transcriptionally by TOX3 via recruiting WDR5 and the subsequent histone H3K4 tri-methylation in CRC, leading to the enhanced self-renewal of cCSCs and drug resistance, particularly chemoresistance, and finally contributing to the recurrence and metastasis of CRC patients after receiving chemotherapy, highlighting the potential of targeted interventing TOX3-WDR5-H3K4me3/ABCG2 signaling axis in overcoming the challenges of drug tolerance and antagonizing CRC recurrence or metastasis, and also the potential of developing TOX3-WDR5/ABCG2 signaling axis as a group of cascade biomarkers for predicting the survival of CRC patients with recurrence or metastasis.

## Materials and methods

### Cell lines

CRC cell lines (RKO, LoVo, DLD1, HCT116) were obtained from American Type Culture Collection (Manassas, Virginia, United States of America). RKO were cultured in Dulbecco’s Modified Eagle’s Medium (DMEM) supplemented with 10% FBS. LoVo, DLD1, and HCT116 were maintained in RPMI-1640 medium containing 10% FBS. All the cell lines mentioned above were cultured in a humidified atmosphere with 5% CO_2_ at 37°C.

### Reagents and antibodies

Oxaliplatin, irinotecan, and regorafenib were purchased from Selleck (S1224, S5026, S1178). Antibodies against GAPDH, TFIIB, CD133(18470-1-AP), CD44 (15675-1-AP), SOX2(11064-1-AP), H3K9me3(61013), H3K27ac(39085), H3K27me3(91404), DPY30(16281-AP), RBBP5(61406), and ABCG2(27286-1-AP) were purchased from Proteintech Group (Wuhan, Hubei, China). The antibodies against EpCAM (#2929), WDR5 (#13105), H3K4me3 (#9751) were purchased from Cell Signaling Technology (USA). The antibodies against Ki67 (sc-23900) and ASH2L (sc-81184) were purchased from Santa Cruz Biotechnology (USA).

### Plasmids and lentivirus infection

The Plvx-IRES-mCherry C1 human ABCG2 plasmid was purchased from YouBio company (Hunan, China). To generate the pcDNA3.1- TOX3 and pcDNA3.1- WDR5 plasmid, we amplified the full-length TOX3 and WDR5 gene by RT-PCR from LOVO cell cDNA and cloned it into pcDNA3.1 vector (V790, Invitrogen, USA). The infection was mediated by lentivirus following the packaging kit (Lentc-Pac HIV expression packaging kit, GeneCopoeia). The transient transfection was performed using Lipofectamine 3000 reagent according to the manufacturer’s protocol (Invitrogen, Carlsbad, California, USA).

### siRNA design and transfection

The siRNAs targeting TOX3 (siRNA1: 5′- GGAUCAGAGCCACACACAATT-3′ and 5′- UUGUGUGUGGCUCUGAUCCTT-3′; siRNA2: 5′- GCGUCGACCAAUCUAACAUTT-3′ and 5′- AUGUUAGAUUGGUCGACGCTT-3′), siRNAs targeting WDR5 (siRNA1: 5′- GCUCAGAGGAUAACCUUGUTT-3′ and 5′- ACAAGGUUAUCCUCUGAGCTT-3′; siRNA2: 5′- CCCAGUCCAACCUUAUUGUTT-3′and 5′- ACAAUAAGGUUGGACUGGGTT-3′), negative control siRNA (5′-UUCUCCGAACGU GUCACGUTT-3′ and 5′-ACGUGACACGUUCGGAGAATT-3′) were purchased from Shanghai GenePharma Co (Shanghai, China). Cells plated in 96-well plates or 6-well plates were transfected with 0.2 or 2 μg of siRNAs for each well using lipo2000 according to the manufacturer’s protocol (Invitrogen, Carlsbad, California, USA).

### Patients cohort and tissue microarrays

In total, 50 pairs of CRC and corresponding metastatic or recurrent tumor tissues were obtained from CRC patients without preoperative treatment in the form of surgical resections collected from 2003 to 2019 at The First Affiliated Hospital of Dalian Medical University (Dalian, China). Among these 50 patients, 30 cases were metastatic tumor tissues and 20 cases were recurrent tumor tissues after radical surgical treatment and chemotherapy. Ethical approval for this retrospective analysis of clinical and biological data was obtained from The First Affiliated Hospital of Dalian Medical University Ethics Committee.

The human CRC tumor tissue microarrays were purchased from Outdo Biotech Company (Shanghai, China). Total 180 cases with CRC were arranged into 3 tissue array blocks. The protein expression levels of TOX3, ABCG2, and WDR5 were analyzed according to the staining level of tissue microarrays. All tissue samples were obtained from the patients without anticancer treatment before tumor resection. Information from all patients such as TNM (tumor-node-metastasis) stage and overall survival was wholly available.

### RNA sequencing and bioinformatics analysis

The cells or tissues were collected, and total RNA was extracted. Expression profiles of mRNA were analyzed using sequencing assay. The edgeR package was used then for DEGs (differential gene statistics) of the 2 groups, and the screening conditions were (Log FC > 2, P Val < 0.05). The top 20 (or 25) with the largest log FC (or sorted according to P Val) were selected to draw heat maps using the heat map package.

### Tumorsphere culture

CRC cells with indicated treatment were digested into single cells and were seeded in 35 mm ultralow attachment dishes (BIOFIL, 2,000 cells/dish) with continuous culture in serum-free DMEM/F12 medium (HyClone) containing B27 and N2 supplement (Gibco), bFGF (20 ng/ml), and EGF (20 ng/ml) for 2 weeks. Then, the pictures of the formed spheroids were taken by inverted microscope (Leica).

### Database analysis

The correlation analysis between TOX3 and ABCG2 in the tumor tissues of CRC patient were conducted in the R2: Genomics Analysis and Visualization Platform (http://r2.amc.nl), and the resulting figures and P values were downloaded. Expression profiles of mRNA data of CRC were downloaded from Gene Expression Omnibus (GEO) database (http://www.ncbi. nlm.nih.gov/geo/), and dataset GSE44076, GSE37182, GSE41258, GSE20916, GSE37182, GSE104645, was used to analyze the expression of TOX3, ABCG2, and WDR5 in normal and CRC. UALCAN analysis (http://ualcan.path.uab.edu/index.html) was used to estimate the expression levels of TOX3 or WDR5 based on cancer stages, sample types, and histological subtype in the Cancer Genome Atlas (TCGA).

### Luciferase reporter assay

To generate the ABCG2 gene promoter-driven luciferase reporter plasmid, the ABCG2 gene upstream sequences (−501 to +360, −381 to +360, −261 to +360, −141 to +360, +1 to +360) were cloned into PGL3-basic plasmid (Promega, Madison, Wisconsin, USA), respectively. CRC cells with different treatments were transfected with pGL3-ABCG2-luciferase plasmids mentioned above and pRL-TK-renilla-luc plasmids. Luciferase activity was measured using a Dual-Luciferase Reporter Assay System (Promega, USA). Data were normalized for transfection efficiency by dividing firefly luciferase activity by Renilla luciferase activity.

### Biotin pull-down assay

The biotin-labeled double-stranded oligonucleotide probes, which correspond to ABCG2 promoter sequence (−861 - +360), were synthesized by PCR using biotin-labeled primers from Sangon Biotech Company. The nuclear proteins (400 μg) were mixed with double-strand biotinylated ABCG2 promoter probe (0.4 μg), streptavidin agarose beads (50 μl) in 500 ml PBSI buffer containing 0.5 mM PMSF, 10 mM NaF, 25 mM β-glycerophosphate, and rotated for 4 h at room temperature (RT). The beads were centrifuged, washed with PBSI buffer for 2 times, and then were resuspended by loading buffer and boiled at 100°C for 10 min. The supernatant was analyzed by western blot.

### Chromatin immunoprecipitation assay (ChIP)

Briefly, the cells were fixed with 1% formaldehyde, scrapped, centrifugated, and sonicated on ice to shear the DNA into the fragments ranging from 200 bp to 500 bp. One-third of the total cell lysate was used as the DNA input control. The remaining two thirds of the lysate were subjected to immunoprecipitations with anti-TOX3, anti-WDR5, anti-H3K4me3, or nonspecific IgG. The immunoprecipitated DNA was subjected to PCR to amplify a 120 bp fragment of ABGC2 promoter. The PCR products were electrophoretically separated on a 2% agarose gel and visualized by GoldView staining.

### MALDI-TOF MS assay

After biotin pull-down assay mentioned above, the complexes are collected and boiled with the loading buffer. The supernatant was loaded onto the SDS/PAGE gel to separate the proteins. After silver staining, the differential bands were cut off and sent to do the mass spectrometry analysis.

### Animal experiments

All animal maintenance and operational procedures were carried out in accordance with the animal license agreement (No.AEE18043) approved by the Animal Care and Ethics Committee of Dalian Medical University. Male BALB/c nude mice, 4 to 5 weeks old, were purchased from Beijing Vital River Laboratory Animal Technology Co. Animals were randomly assigned to different groups when they reached 5 to 6 weeks old and received the corresponding treatments. The animal models were divided into 5 parts:

Part 1. HCT116 cells, including monolayer CRC cells and spheroids, were injected subcutaneously into the right flank of each mouse, respectively.

Part 2. HCT116 cells with stable knockdown of TOX3 and/or stable overexpression of ABCG2 and the corresponding control cells were firstly established by lentivirus infection. Then, the mice were randomly divided into 4 groups (4 mice per group): Scr+GFP, TOX3 shRNA+GFP, Scr+OE-ABCG2, TOX3 shRNA+OE-ABCG2, and the established stable cells (1 × 10^7^ suspended in 100 μl PBS) were injected subcutaneously into the right flank of each mouse, respectively, and LDA assay were performed.

Part 3. The same treatments were done as part 2. Immediately after tumor formation, the mice were i.p. administered oxaliplatin (4 mg/kg) once every 48 h for 14 days and LDA assay were performed.

Part 4. LOVO cells with stable overexpression of TOX3 or not (LOVO-GFP or LOVO-OE TOX3) were injected subcutaneously into the right flank of Male Balb/c nude mice for LDA assay. Immediately after tumor formation, mice were i.p. administered OICR-9429 (1.5 mg/kg per day) or vehicle (dimethylsulfoxide/distilled water at 1:24) for 7 days.

Part 5. The same treatments were operated as part 4. Immediately after tumor formation, besides OICR-9429, oxaliplatin (4 mg/kg) was employed to treat the nude mice through i.p. administration once every 48 h for 14 days. During the treatment, tumor diameter was recorded using digital calipers and the tumor volume in mm^3^ was calculated as V = (width^2^ × length)/2. Meanwhile, the number of tumors was counted. At the final time point, the mice were killed by cervical dislocation, and the tumors were removed, weighed, and partial of them in each group were quickly tripsinized for colony and spheroid formation assay or frozen in liquid nitrogen for WB analysis, while the rest of them were then fixed in 10% formalin used for the IHC experiment.

### Induction of CRC in situ model in mice

For the AOM/DSS-induced CRC model, BALb/c mice were injected intraperitoneally with 10 mg of AOM (Sigma) per kg body weight. Five days later, 3% DSS (molecular mass 40 kDa; Aladdin, China) was given in the drinking water for 6 days followed by regular drinking water for 2 weeks. This cycle was repeated twice with 2.5% DSS. On day 10, to induce drug resistance, oxaliplatin (2 mg/kg) or NS (normal saline) induction was performed. The mice were killed on day 90, and partial fresh tumor tissues were digested to get the generations of CRC cell spheroids, partial were used for western blot assay, and the rest were fixed in 10% formalin used for the IHC assay.

### Western blot

Proteins from cell lysate were quantified using a BCA protein assay kit, separated by electrophoresis in a 10% polyacrylamide gel (SDS-PAGE) and electrophoretically transferred to a polyvinylidenefluoride membrane (Millipore, USA). The samples were probed with the target-specific antibodies. The protein bands were detected finally by enhanced chemiluminescence.

### Cell viability assay

Cell viability was determined using the MTT assay. Briefly, different cell lines were seeded into 96-well plates with continuous culture for 12 to 24 h, and then the culture medium was changed to fresh, and the cells were treated accordingly. Five replicated wells were put up for each group. The effect of treatment on cell viability was assessed by comparison to the control group.

### FACS assay

FACS assay was performed according to the protocol reported in the previous study [29]. Briefly, cells seeded in plates were treated accordingly, and 48 h later, the cells were harvested and resuspended in binding buffer and stained using Annexin V-FITC/PI staining kit (Key-Gene BioTech, Nanjing, Jiangsu, China) based on the producers’ guidelines. The number of apoptotic cells was analyzed by flow cytometry (BD ACCURI C6).

### Colony formation assay

Briefly, different CRC cell lines were seeded into 6-well plate (2 × 10^3^ per well) and cultured for 12 to 24 h. Then, the cells were treated accordingly. After 24 or 48 h, the medium was changed into fresh containing 10% FBS and the cells were incubated for another 2 weeks until they grew into macroscopic colonies. Finally, the medium was removed, and the colonies were stained by 0.1% crystal violet and counted.

### Immunofluorescence assay

For confocal microscopy analysis, cells growing on chamber slides were washed by PBS and fixed for 10 min at RT with 4% paraformaldehyde. The samples were permeabilized with 0.2% Triton X-100 for 5 min, and then blocked with 10% bovine serum albumin (BSA) in PBS for 30 min. Antibodies were added to the sample and incubated overnight at 4°C. Following 10-min washes each time for 3 times with PBS, fluorescein isothiocyanate or rhodamine-conjugated secondary antibodies were added in 1% blocking solutions and incubated for 90 min. Subsequently, the stained samples were mounted with vectashield solution containing 4′6-diamidino-2-phenylindole (DAPI) to stain cell nuclei. After 5 additional 10-min washes, the samples were examined with a Leica DM 14000B confocal microscope.

### Co-immunoprecipitation

Briefly, cell nuclear extract proteins (400 μg) were incubated respectively with a specific antibody against the target protein or a nonspecific IgG at a final concentration of 5 μg/ml for 12 h at 4°C. The immune complexes were pulled down by protein A/G agarose beads (Santa Cruz Biotechnology). After extensive washing with cold PBS, the immunoprecipitated proteins were separated by SDS-PAGE and analyzed by western blotting using specific antibody.

### Immunohistochemistry staining

Tumors were dissected and fixed in 10% formalin overnight. Then, the fixed tissues were embedded in paraffin for sectioning and incised to 4 μm thick. After antigen retrieval in hot citrate buffer (pH 6.0) for 20 min, immunohistochemistry was performed according to the DAB (3, 30-diaminobenzidine) Kit (Origene, China). Sections were stained with hematoxylin to recognize nuclear.

### Limiting dilution assay

Different CRC cell lines or CRC cells digested from the tumor xenografts in mouse were seeded into 6-well ultralow attachment culture plate (50, 100, 200 cells per well) in DMEM/F12 medium (Gibco) containing B27 and N2 supplement (Gibco), bFGF (20 ng/ml) and EGF (20 ng/ml) for 2 weeks, and then the proportion of the formed CSCs was calculated using ELDA (Extreme Limiting Dilution Analysis) software.

### Ethics approval and consent to participate

The animal research was approved by the Animal Experimental Ethical Committee of Dalian Medical University (Liaoning, China) and the study was carried out in strict accordance with the recommendations in the Guide for the Care and Use of Laboratory Animals approved by the Committee. The human tumor tissue research was approved by the Biomedical Ethics Committee of Dalian Medical University (Liaoning, China) with the approval No. ChiCTR1900028751.

### Statistical analysis

Data are represented as mean ± standard deviation (SD). To compare the statistical differences, SPSS software (version 11.0, Chicago, Illinois, USA) or Graph Pad Prism software (San Diego, California, USA) was used by two-tailed Student’s *t* test or one-way ANOVA as approximate. *P* < 0.05 was considered to be a statistically significant difference.

## Supporting information

S1 Fig ABCG2 was highly expressed and transcriptionally regulated by TOX3 in cCSCs. (TIF)

S2 Fig TOX3 was highly expressed in CRC and promoted cCSCs expansion. (TIF)

S3 Fig Stable knockdown of TOX3 inhibited cCSCs expansion. (TIF)

S4 Fig TOX3 sensitizes CRC cells to the treatment of chemotherapeutics and targeted agent. (TIF)

S5 Fig TOX3 sensitizes CRC cells to the treatment of chemotherapeutics and targeted agent by targeting ABCG2. (TIF)

S6 Fig WDR5 was highly expressed in CRC and anchored at the ABCG2 promoter in cCSCs. (TIF)

S7 Fig WDR5 regulated ABCG2 expression and promoted cCSCs expansion. (TIF)

S8 Fig TOX3 synergized with WDR5 to co–promote cCSCs expansion and drug resistance in vivo and in vitro. (TIF)

S9 Fig The high expression of ABCG2 was positively correlated with TOX3 and WDR5 expression in CRC tissues and predicted poor prognosis in patients with CRC. (TIF)

S1 Data Underlying data for Figs 1–7. (XLSX)

S2 Data Underlying data for S1–S9 Figs. (XLSX)

S1 Raw Images Original scan images for Figs 1C–1E, 1H, 2A, 2E, 2K, 4A, 4C, 4E, 4G, 5B, 5G, 6G, S1B, S1H, S2G, S3A, S3E, S6E, S6G and S7E. (PDF)

S1 Table Information of 20 down–regulated genes from RNA–seq (DOCX). (DOCX)

S2 Table Information of 20 up–regulated genes from RNA–seq. (DOCX)

S3 Table Correlation analyses of gene expression in relation to clinicopathologic variables from 94 patients with colorectal cancer. (DOCX)

S4 Table Five–year overall survival rate analysis by Kaplan–Meier for 94 patients with colorectal cancer. (DOCX)

S5 Table Univariate analysis of Cox’s proportional hazards regression model for 94 patients with colorectal cancer. (DOCX)

S6 Table Correlation analyses of gene expression in relation to clinicopathologic variables from 50 patients with colorectal cancer before recurrence. (DOCX)

S7 Table Correlation analyses of gene expression in relation to clinicopathologic variables from 50 patients with colorectal cancer after recurrence. (DOCX)

S8 Table Five–year disease–free survival rate analysis by Kaplan–Meier for 50 patients with colorectal cancer. (DOCX)

S9 Table Univariate analysis of Cox’s proportional hazards regression model for 50 patients with colorectal cancer. (DOCX)

## Abbreviations

BSA: bovine serum albumin
cCSC: colorectal cancer stem cell
CRC: colorectal cancer
CSC: cancer stem cell
DMEM: Dulbecco’s Modified Eagle’s Medium
GEO: Gene Expression Omnibus
LDA: limited dilution assay
RT: room temperature
SD: standard deviation
SNP: single-nucleotide polymorphism
SP: side population
TNM: tumor-node-metastasis
WDR5: WD Repeat Domain 5
