# Supplementary figures and images for: A novel TOX3-WDR5-ABCG2 signaling axis regulates the progression of colorectal cancer by accelerating stem-like traits and chemoresistance
Source: PLoS Biol. 2023 Sep 14;21(9):e3002256. doi: 10.1371/journal.pbio.3002256 (PMC10501593; doi:10.1371/journal.pbio.3002256)

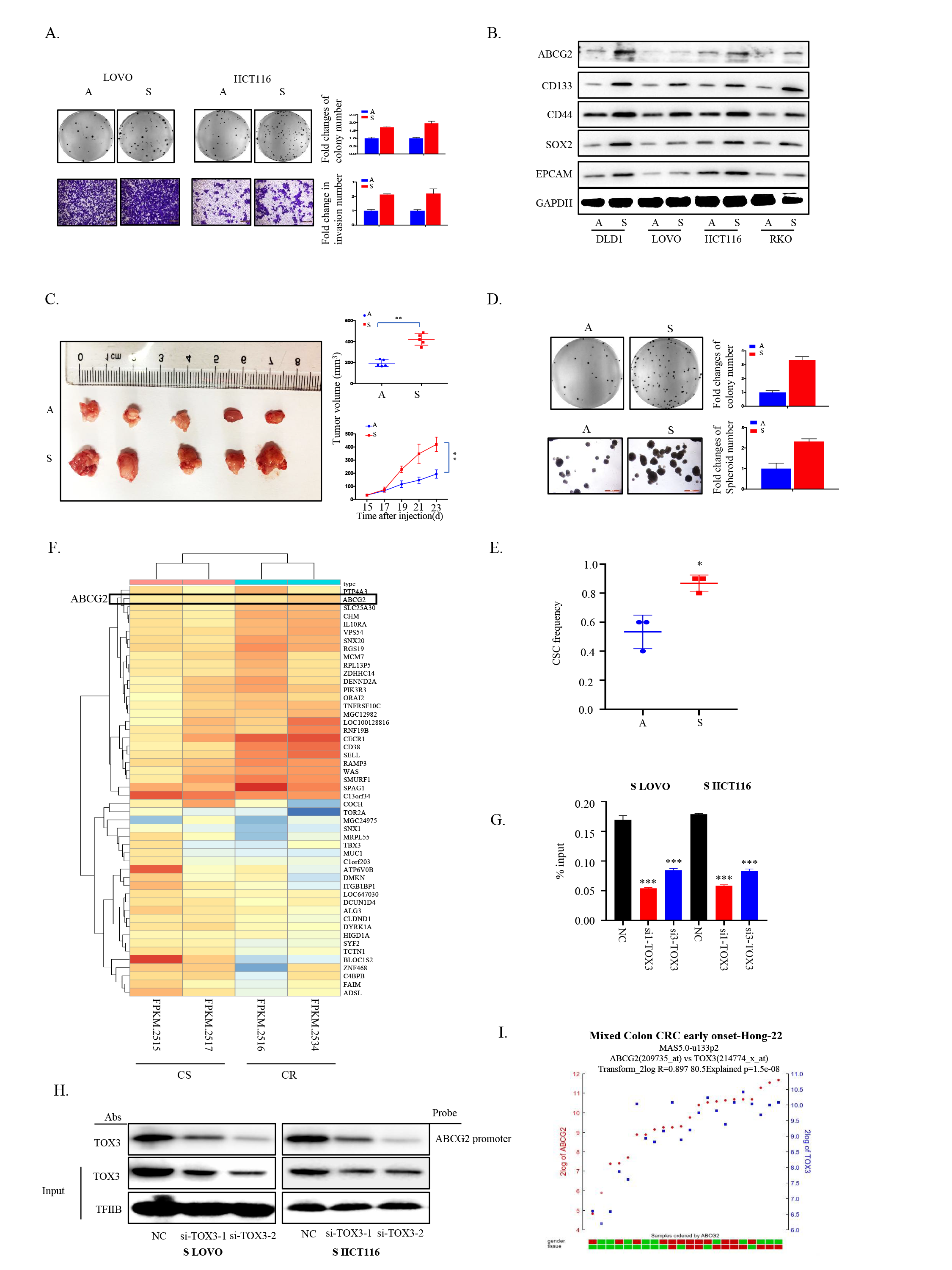

Supplement: S1 Fig — (TIF) [file pbio.3002256.s001.tif]

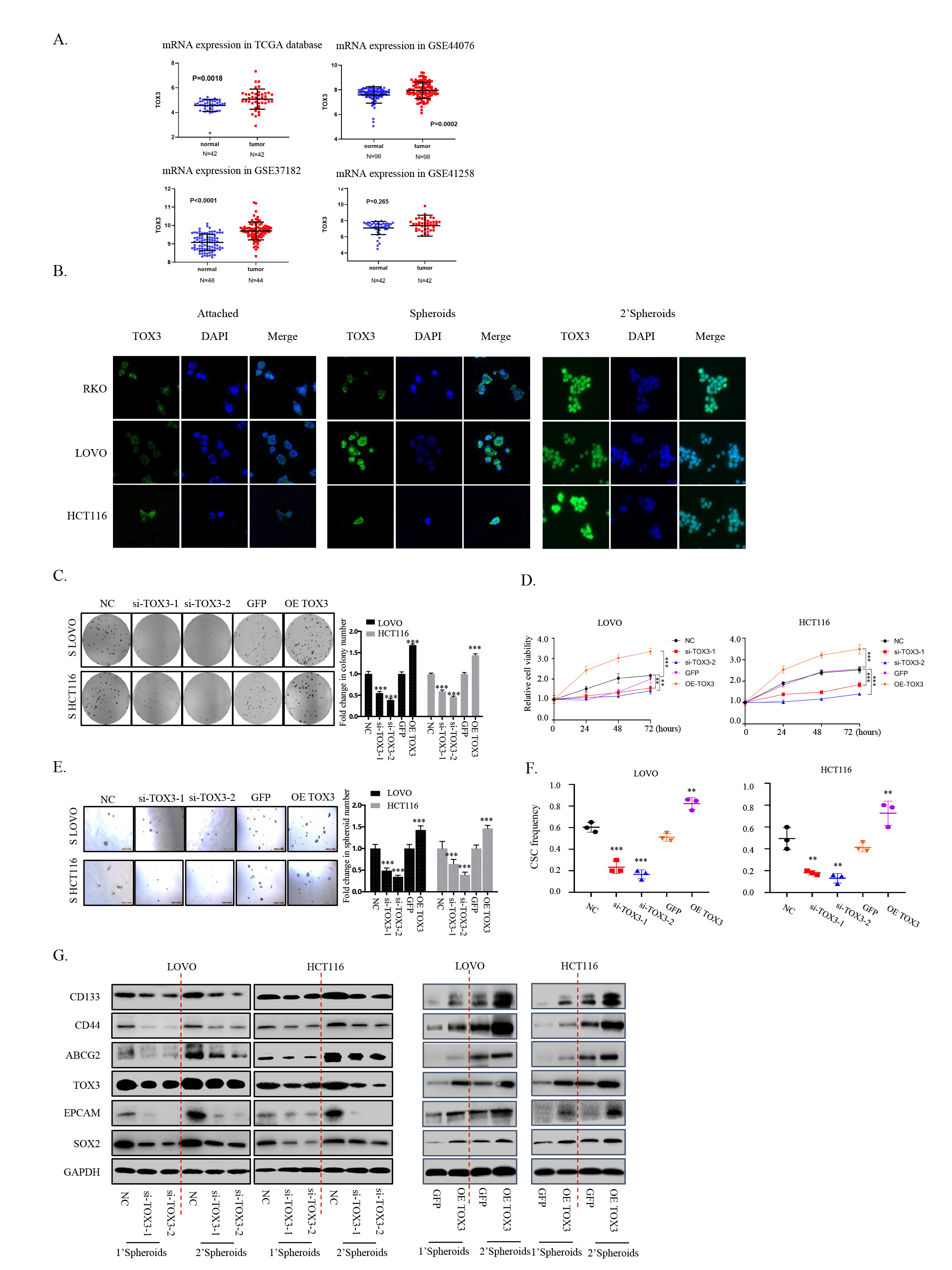

Supplement: S2 Fig — (TIF) [file pbio.3002256.s002.tif]

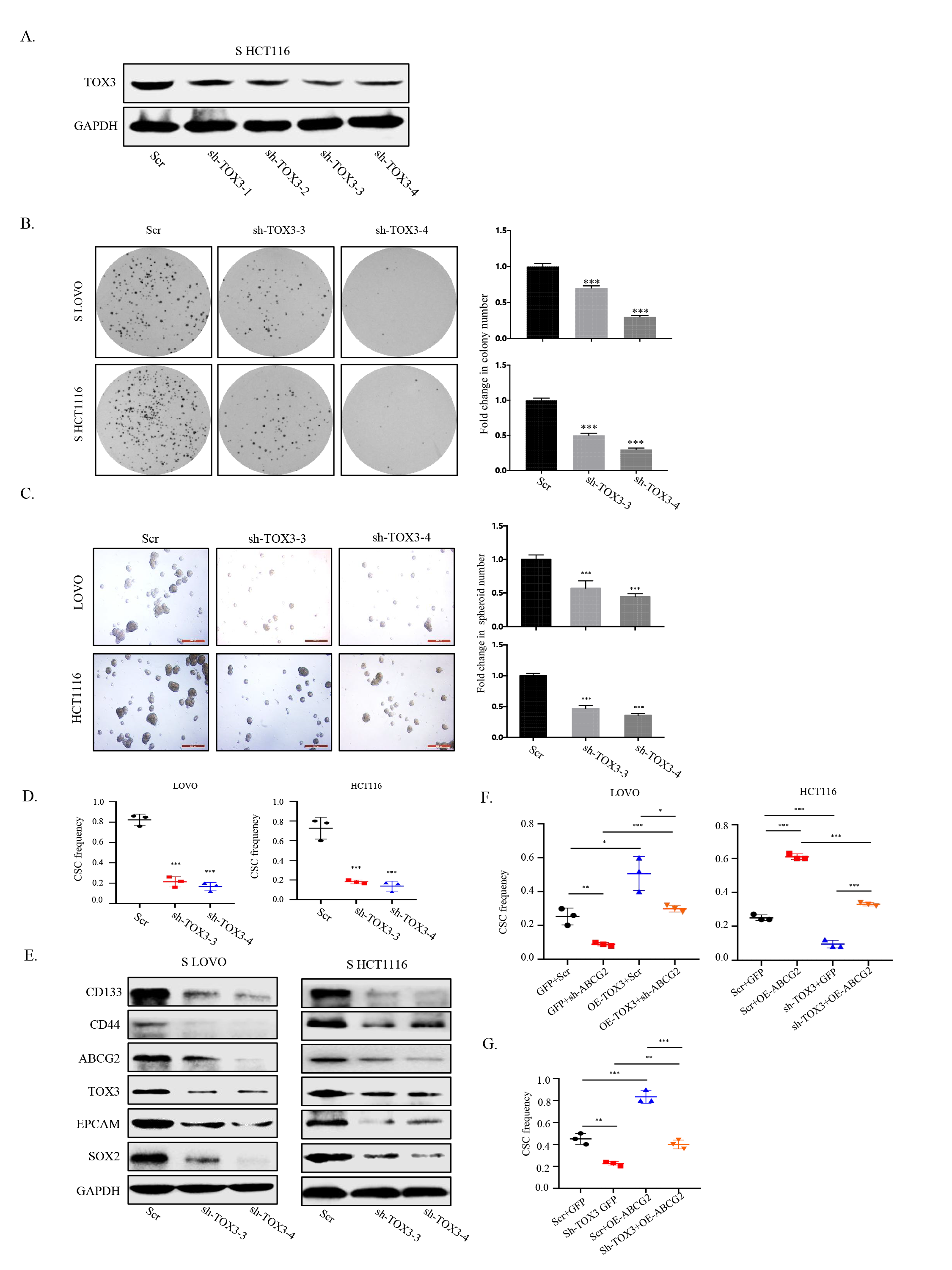

Supplement: S3 Fig — (TIF) [file pbio.3002256.s003.tif]

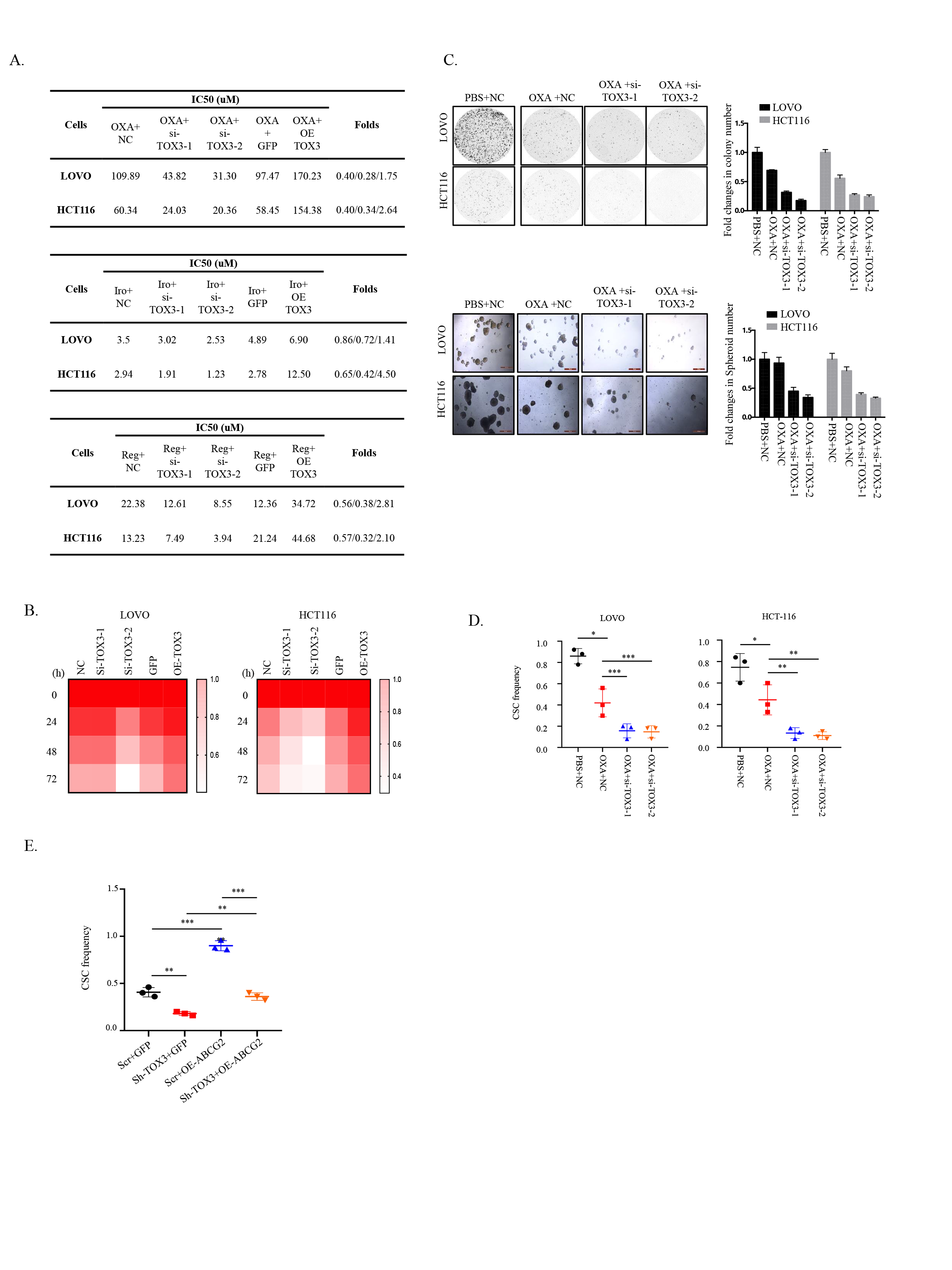

Supplement: S4 Fig — (TIF) [file pbio.3002256.s004.tif]

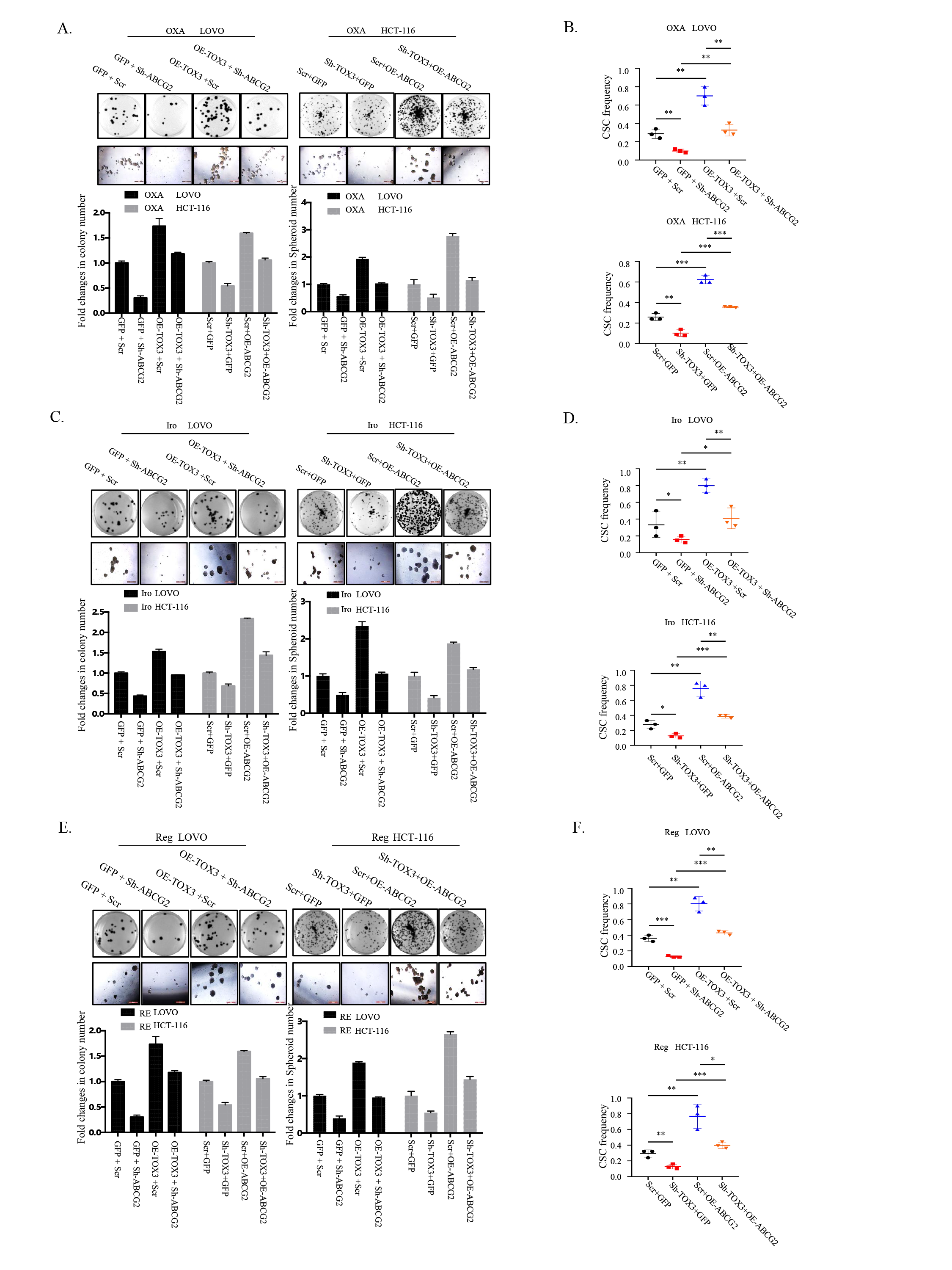

Supplement: S5 Fig — (TIF) [file pbio.3002256.s005.tif]

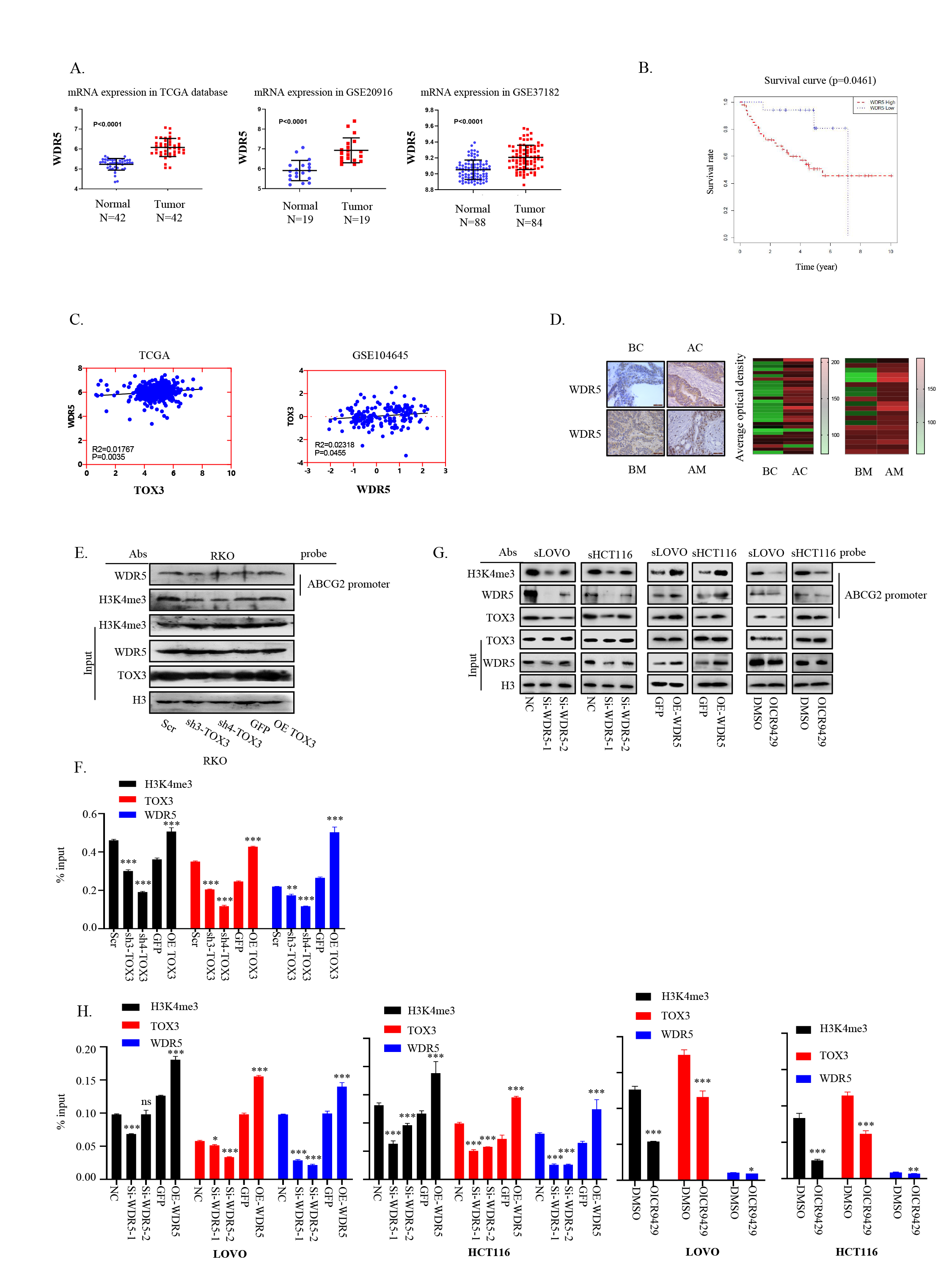

Supplement: S6 Fig — (TIF) [file pbio.3002256.s006.tif]

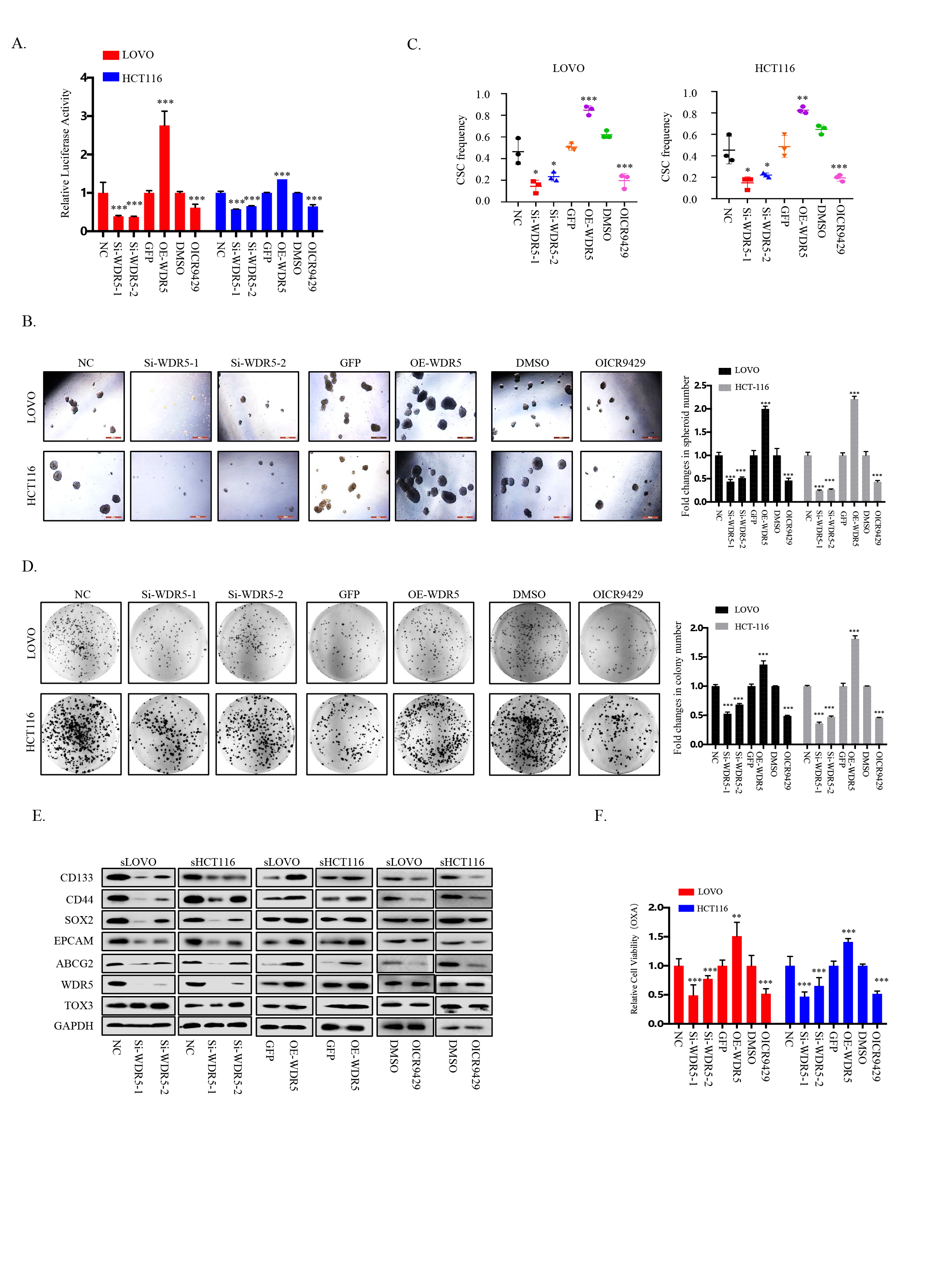

Supplement: S7 Fig — (TIF) [file pbio.3002256.s007.tif]

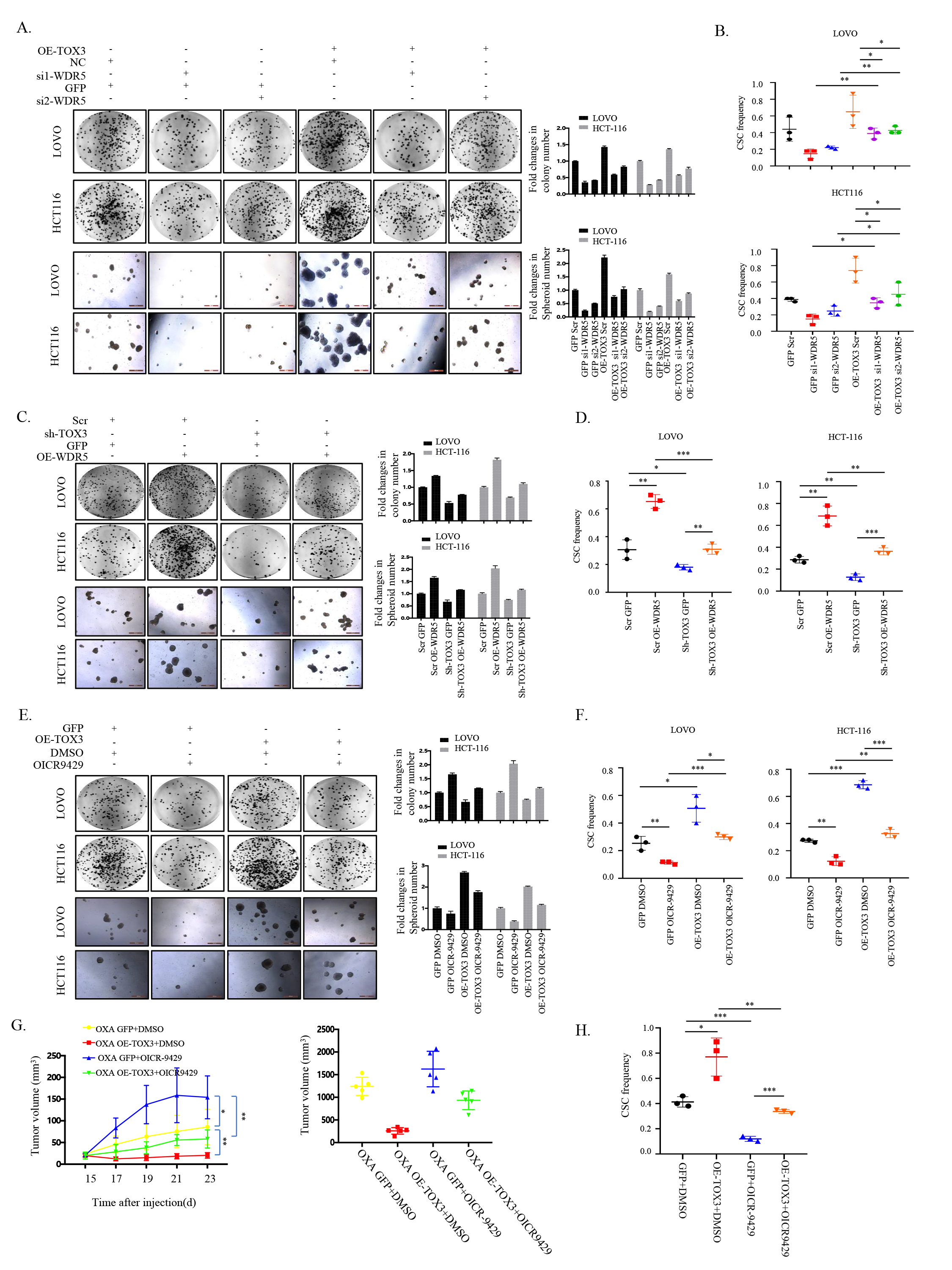

Supplement: S8 Fig — (TIF) [file pbio.3002256.s008.tif]

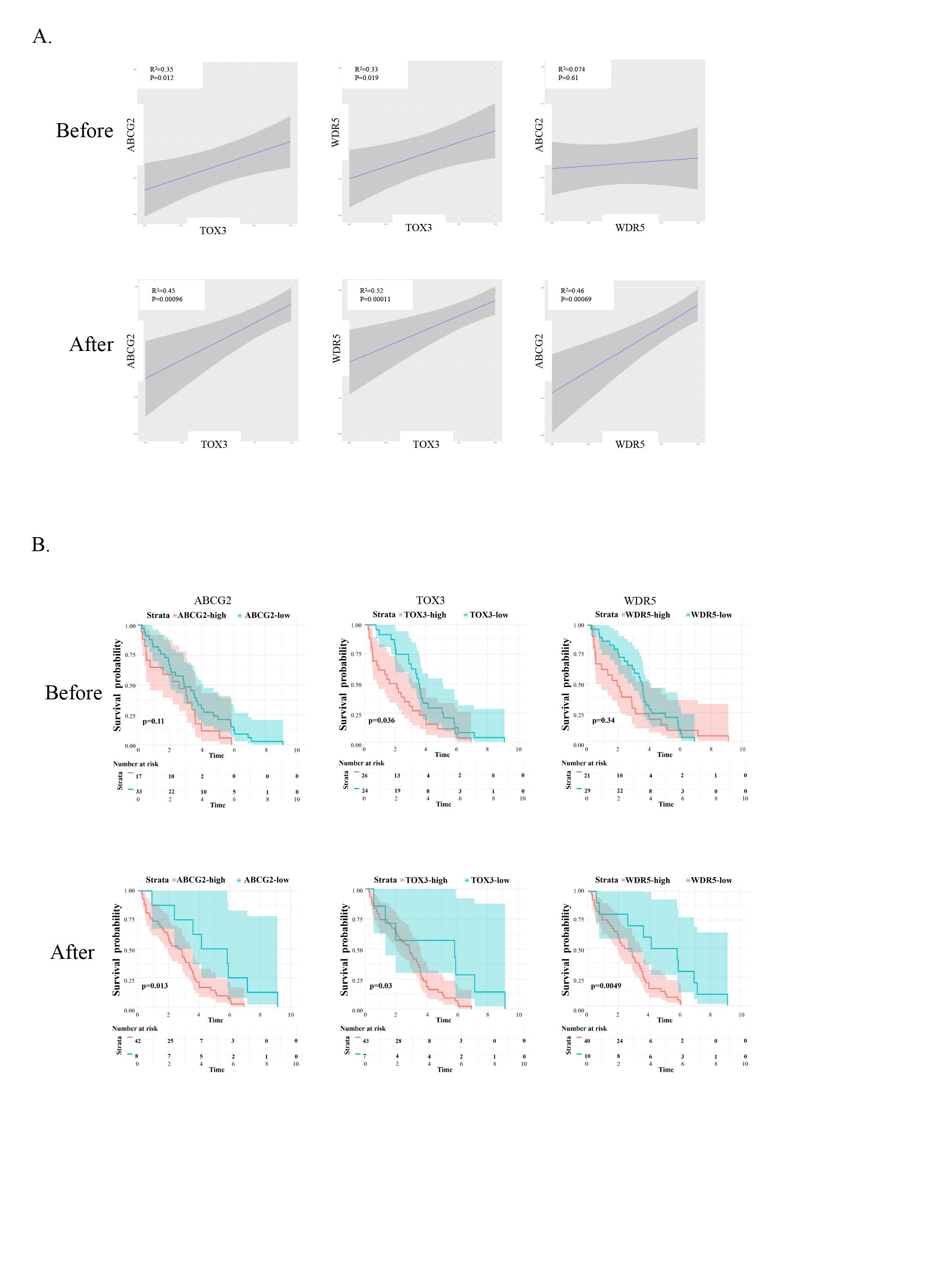

Supplement: S9 Fig — (TIF) [file pbio.3002256.s009.tif]
