# Supplementary material for: A novel TOX3-WDR5-ABCG2 signaling axis regulates the progression of colorectal cancer by accelerating stem-like traits and chemoresistance
Source: PLoS Biol. 2023 Sep 14;21(9):e3002256. doi: 10.1371/journal.pbio.3002256 (PMC10501593; doi:10.1371/journal.pbio.3002256)

F1

F1C

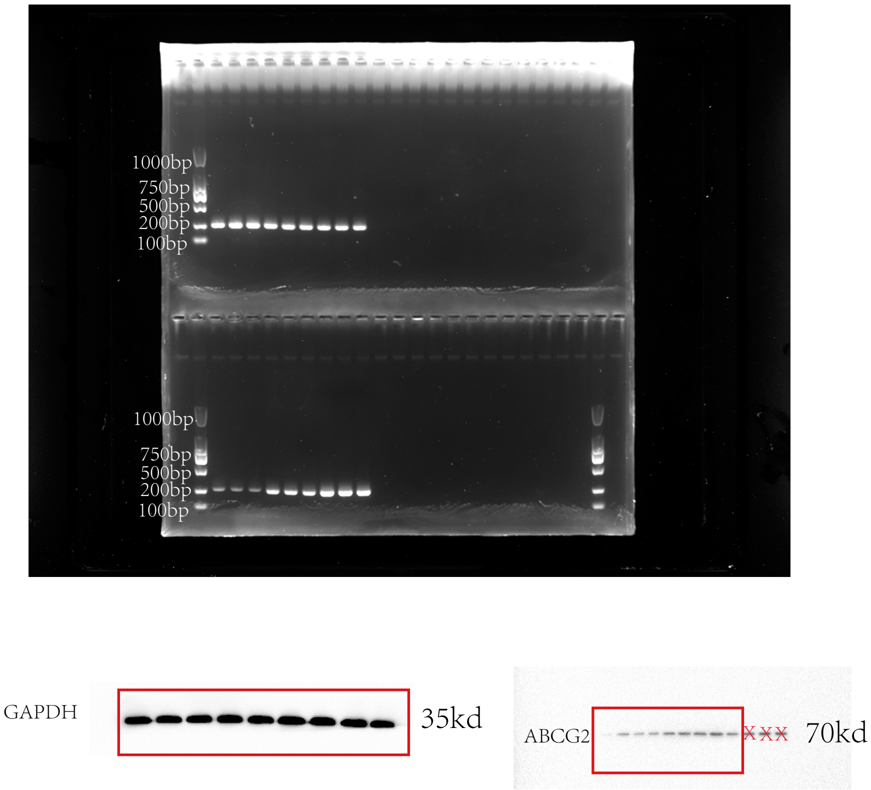

F1D

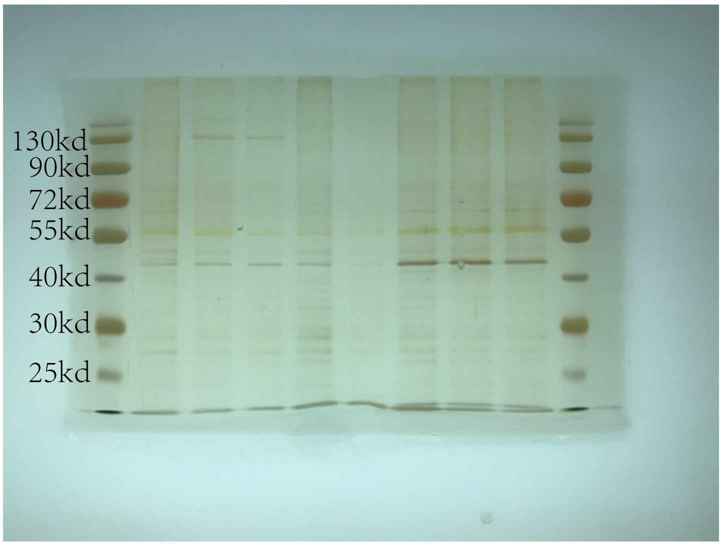

F1E

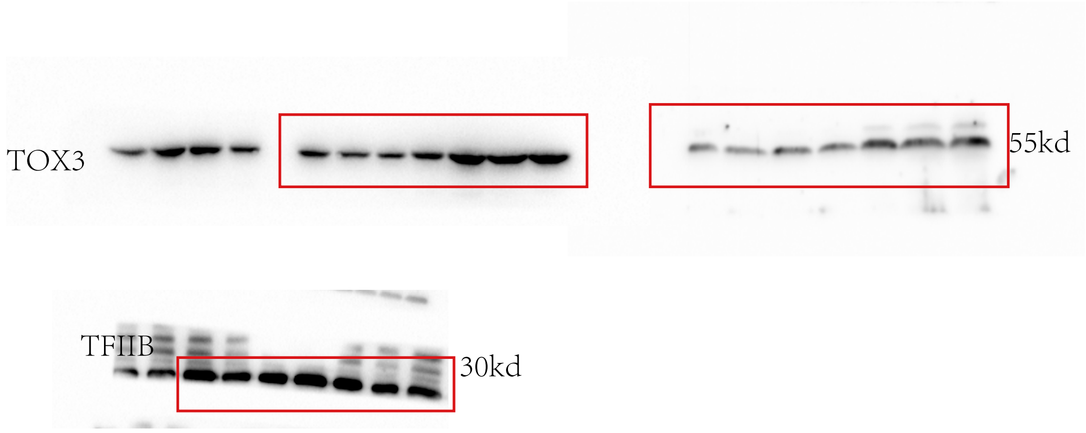

F1

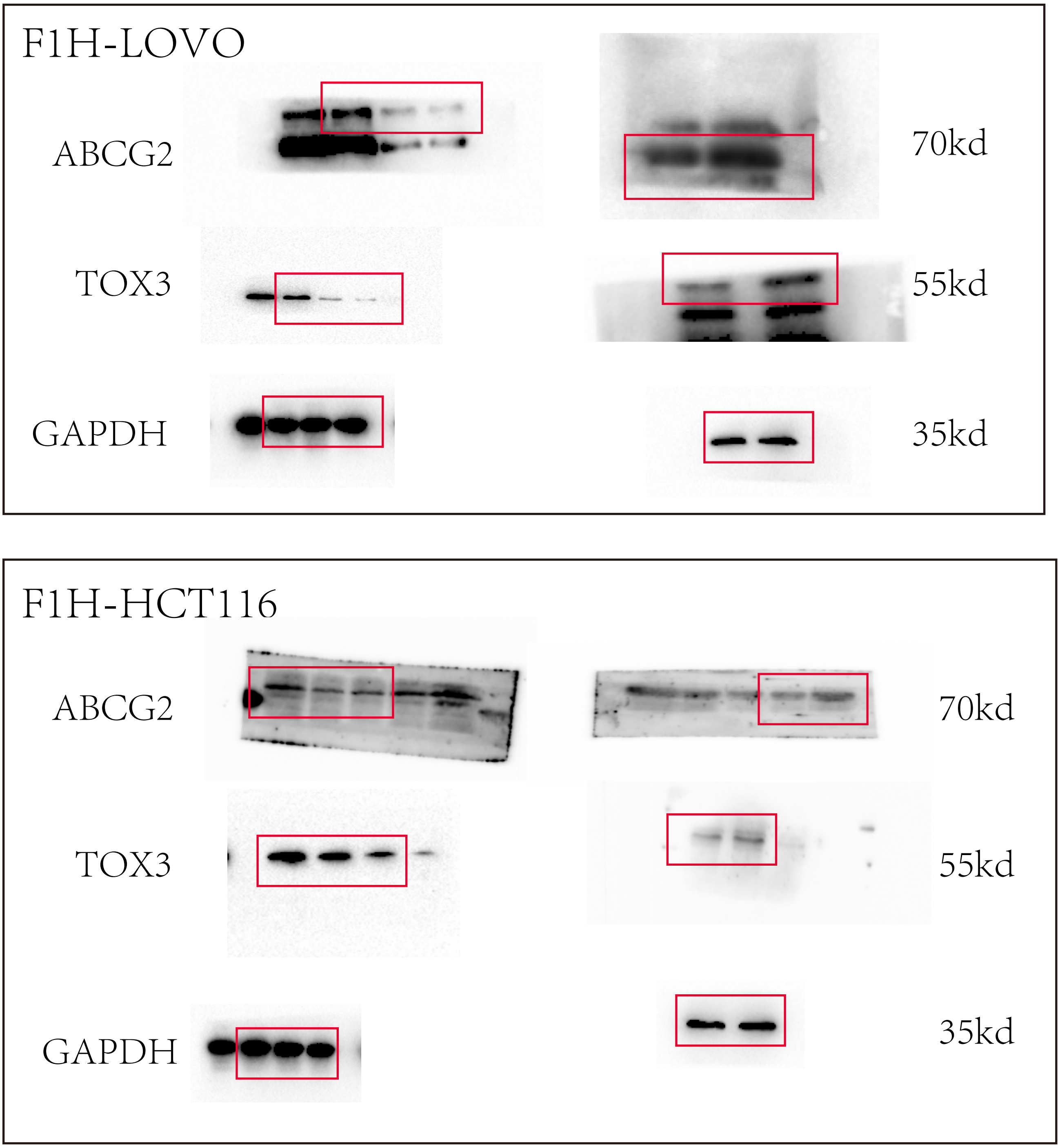
F1H-LOVO
ABCG2
70kd
TOX3
55kd
GAPDH
35kd
F1H-HCT116
ABCG2
70kd
TOX3
55kd
GAPDH
35kd

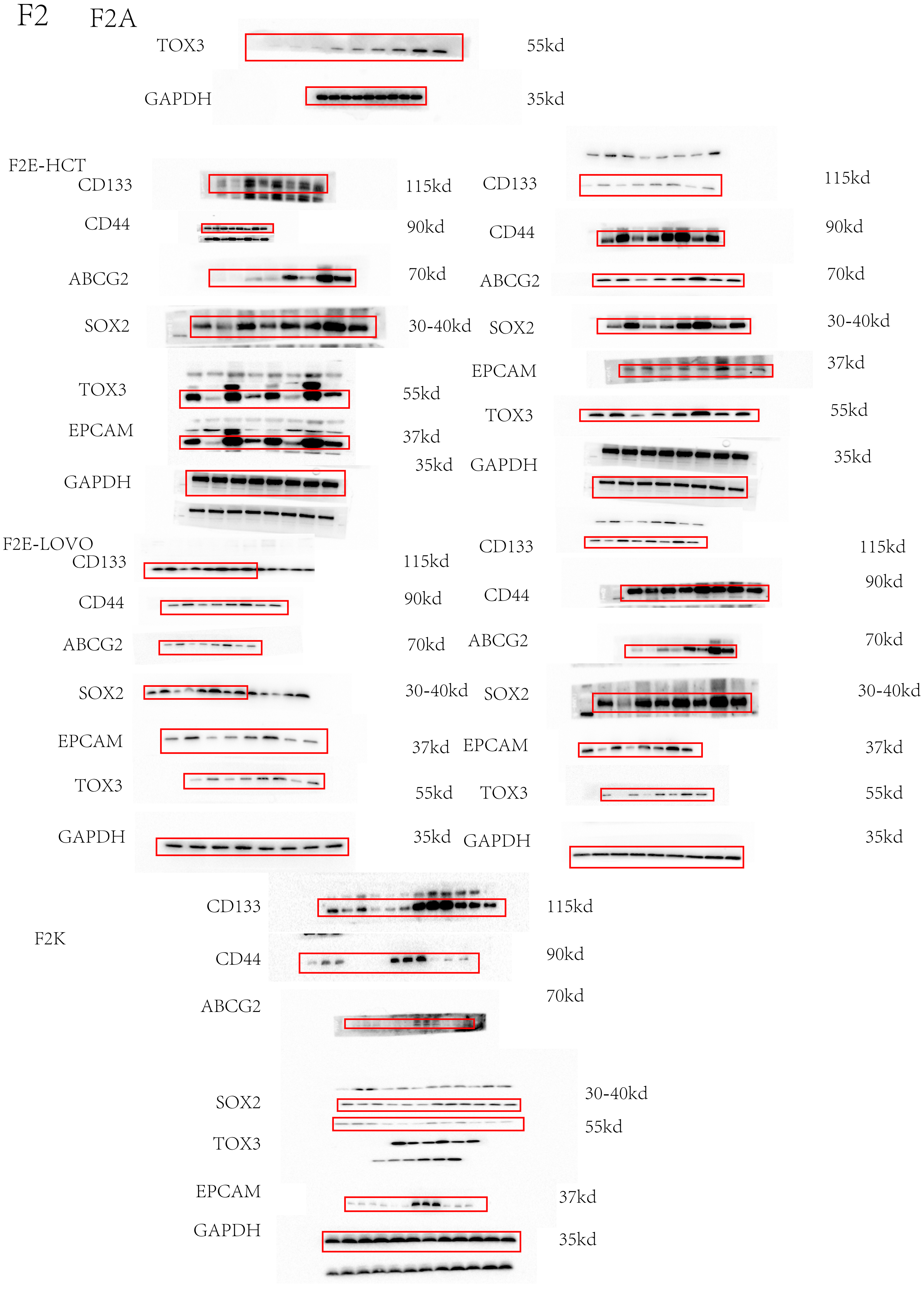
F2
F2A
TOX3
55kd
GAPDH
35kd
F2E-HCT
CD133
115kd
CD44
90kd
ABCG2
70kd
SOX2
30-40kd
TOX3
55kd
EPCAM
37kd
GAPDH
35kd
CD133
115kd
CD44
90kd
ABCG2
70kd
SOX2
30-40kd
EPCAM
37kd
TOX3
55kd
GAPDH
35kd
F2E-LOVO
CD133
115kd
CD44
90kd
ABCG2
70kd
SOX2
30-40kd
EPCAM
37kd
TOX3
55kd
GAPDH
35kd
CD133
115kd
CD44
90kd
ABCG2
70kd
SOX2
30-40kd
EPCAM
37kd
TOX3
55kd
GAPDH
35kd
F2K
CD133
115kd
CD44
90kd
ABCG2
70kd
SOX2
30-40kd
TOX3
55kd
EPCAM
37kd
GAPDH
35kd

F4

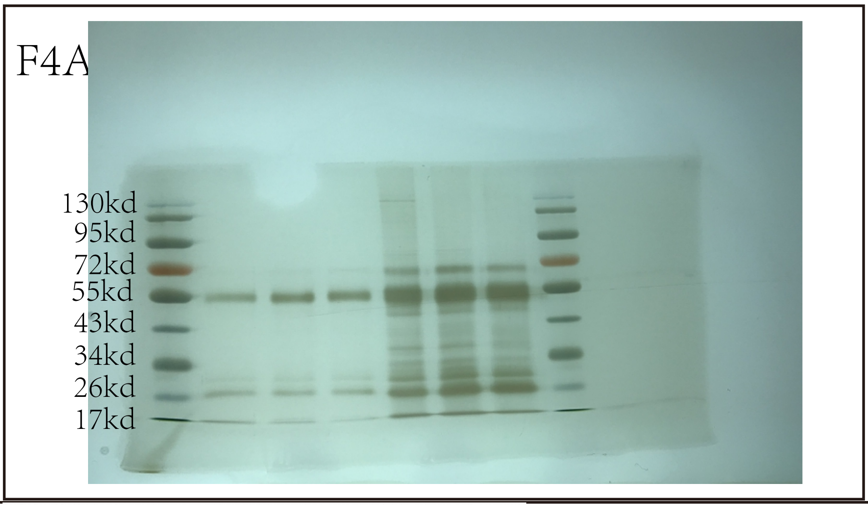
F4A
130kd
95kd
72kd
55kd
43kd
34kd
26kd
17kd

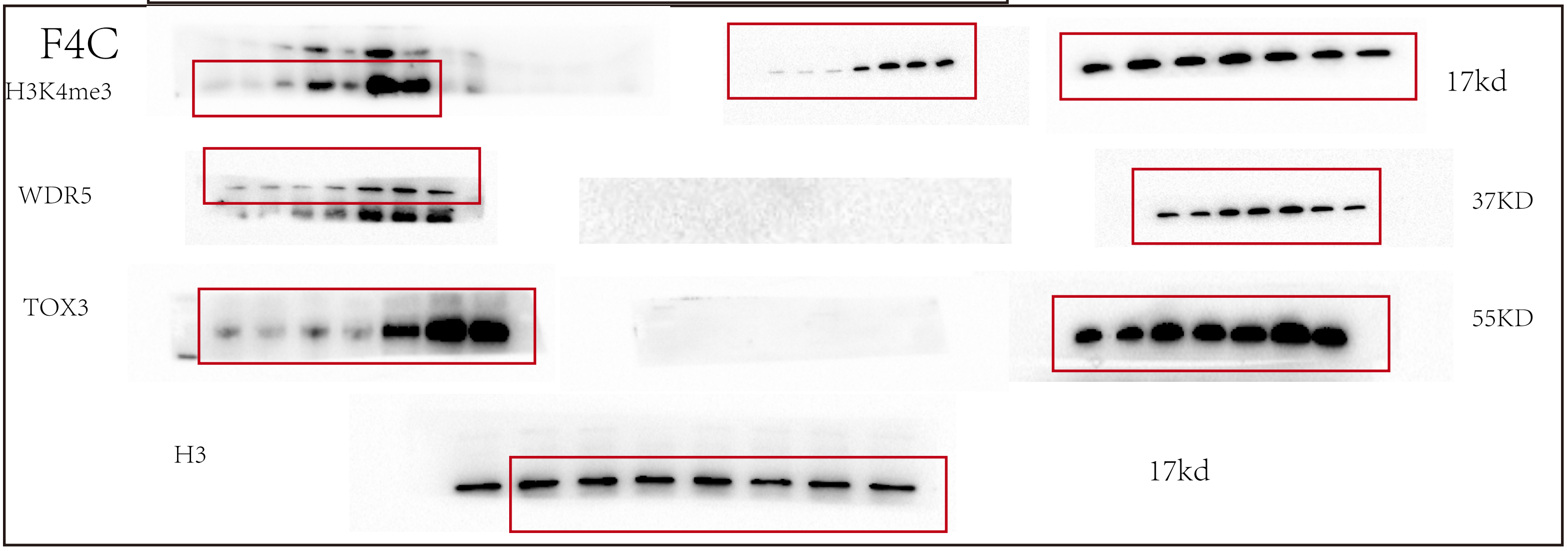
F4C
H3K4me3
17kd
WDR5
37KD
TOX3
55KD
H3
17kd

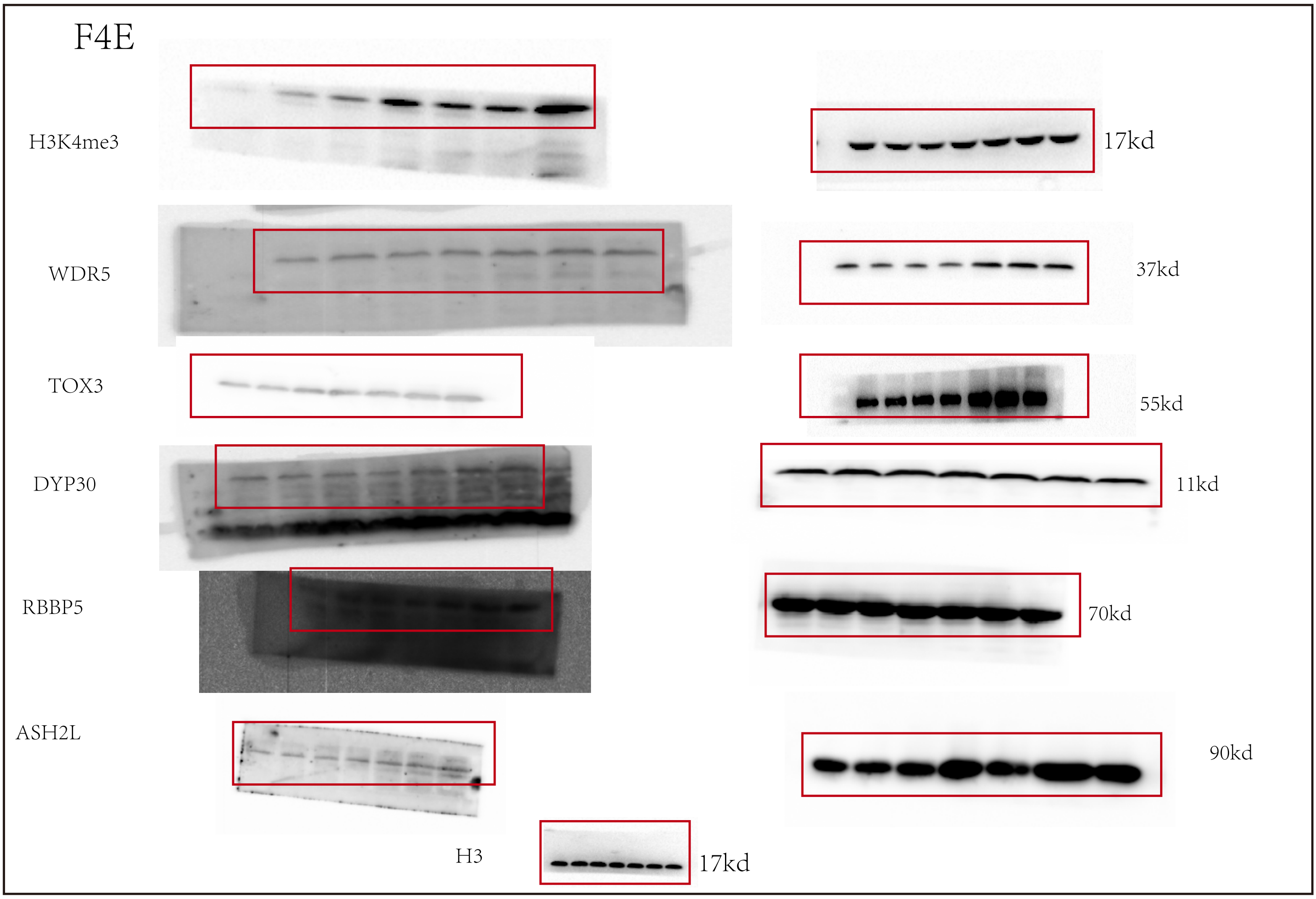
F4E
H3K4me3
17kd
WDR5
37kd
TOX3
55kd
DYP30
11kd
RBBP5
70kd
ASH2L
90kd
H3
17kd

F4

F4G-HCT116

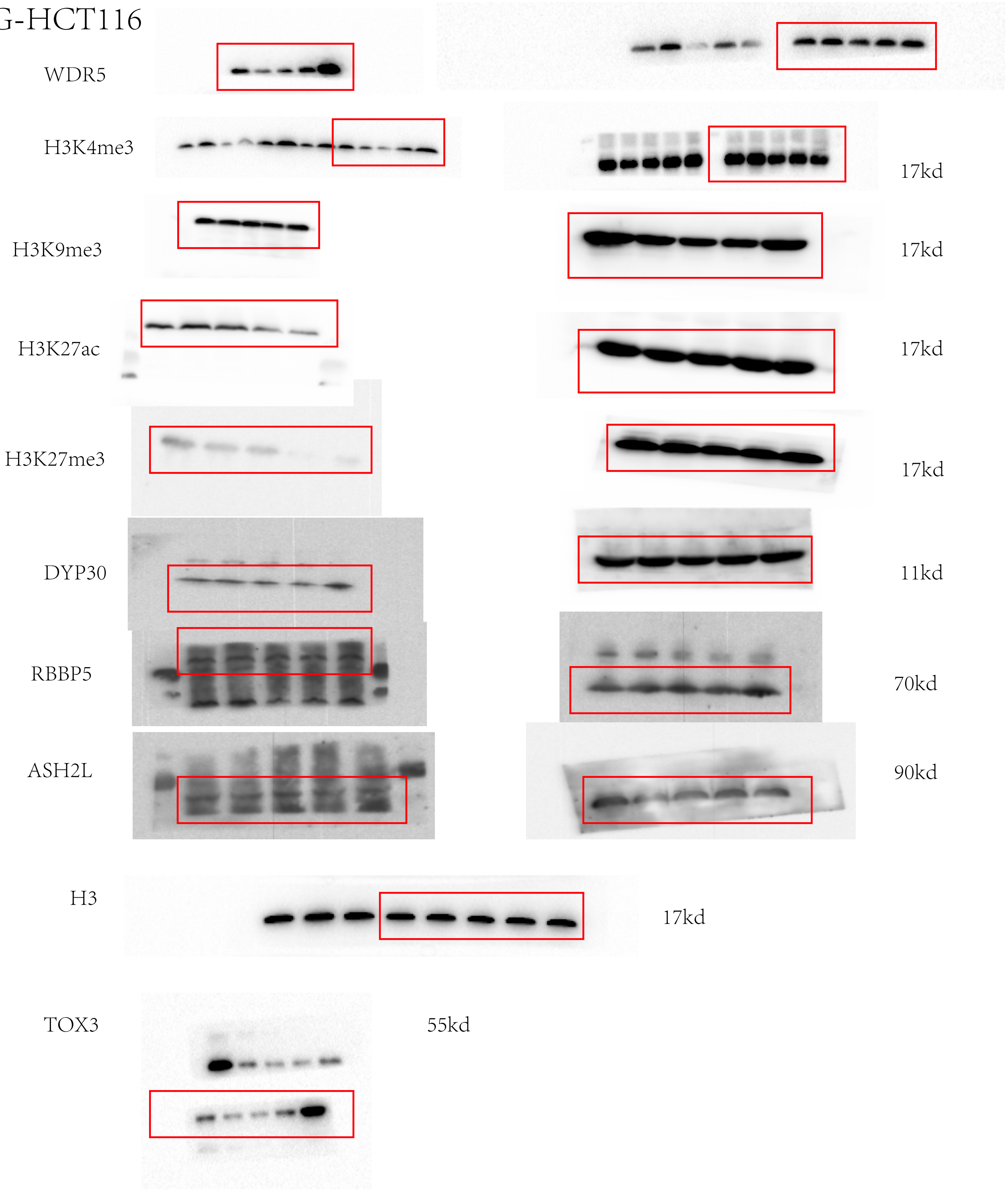

F4

F4G-LOVO

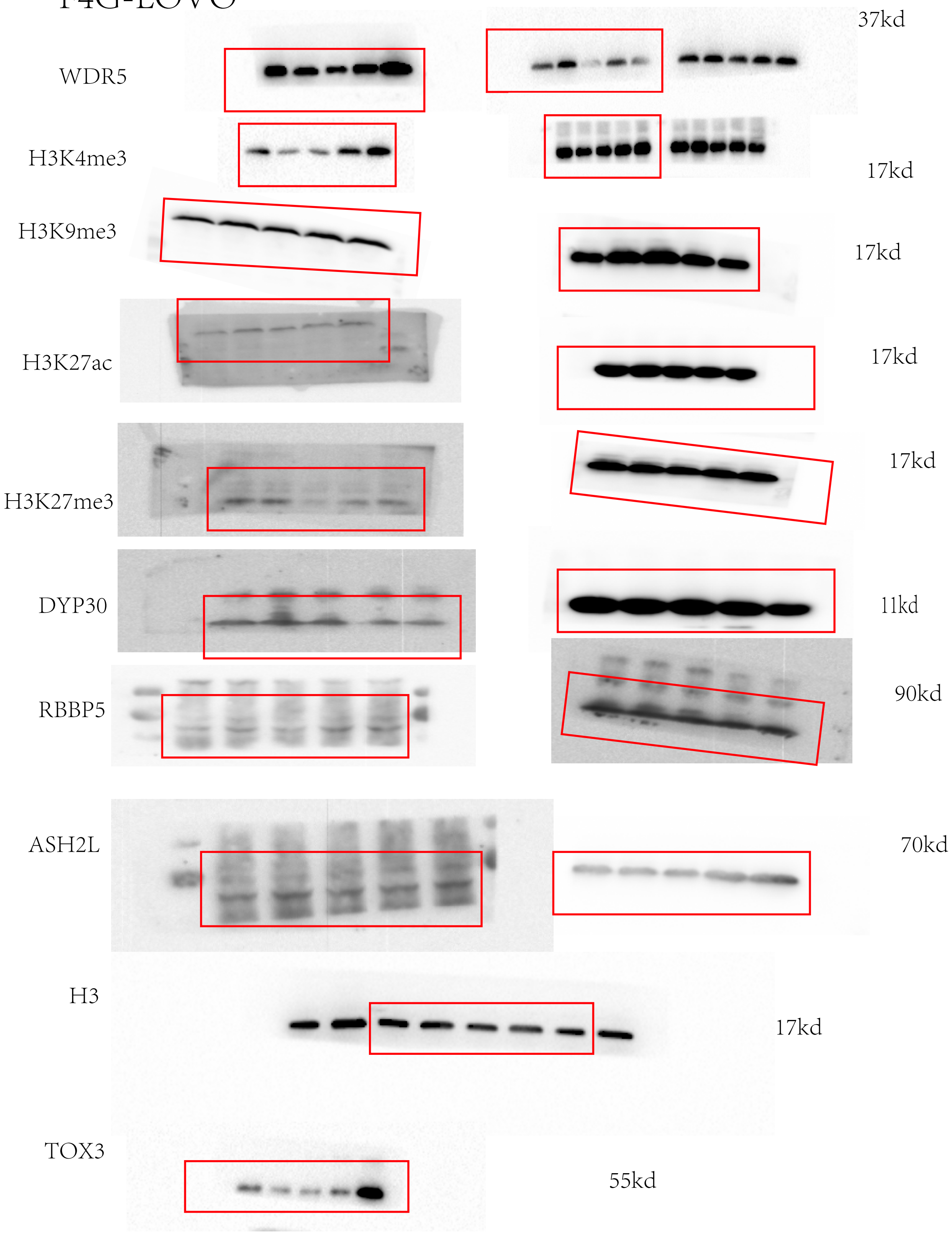
WDR5
37kd
H3K4me3
17kd
H3K9me3
17kd
H3K27ac
17kd
H3K27me3
17kd
DYP30
11kd
RBBP5
90kd
ASH2L
70kd
H3
17kd
TOX3
55kd

F5

F5B-HCT116

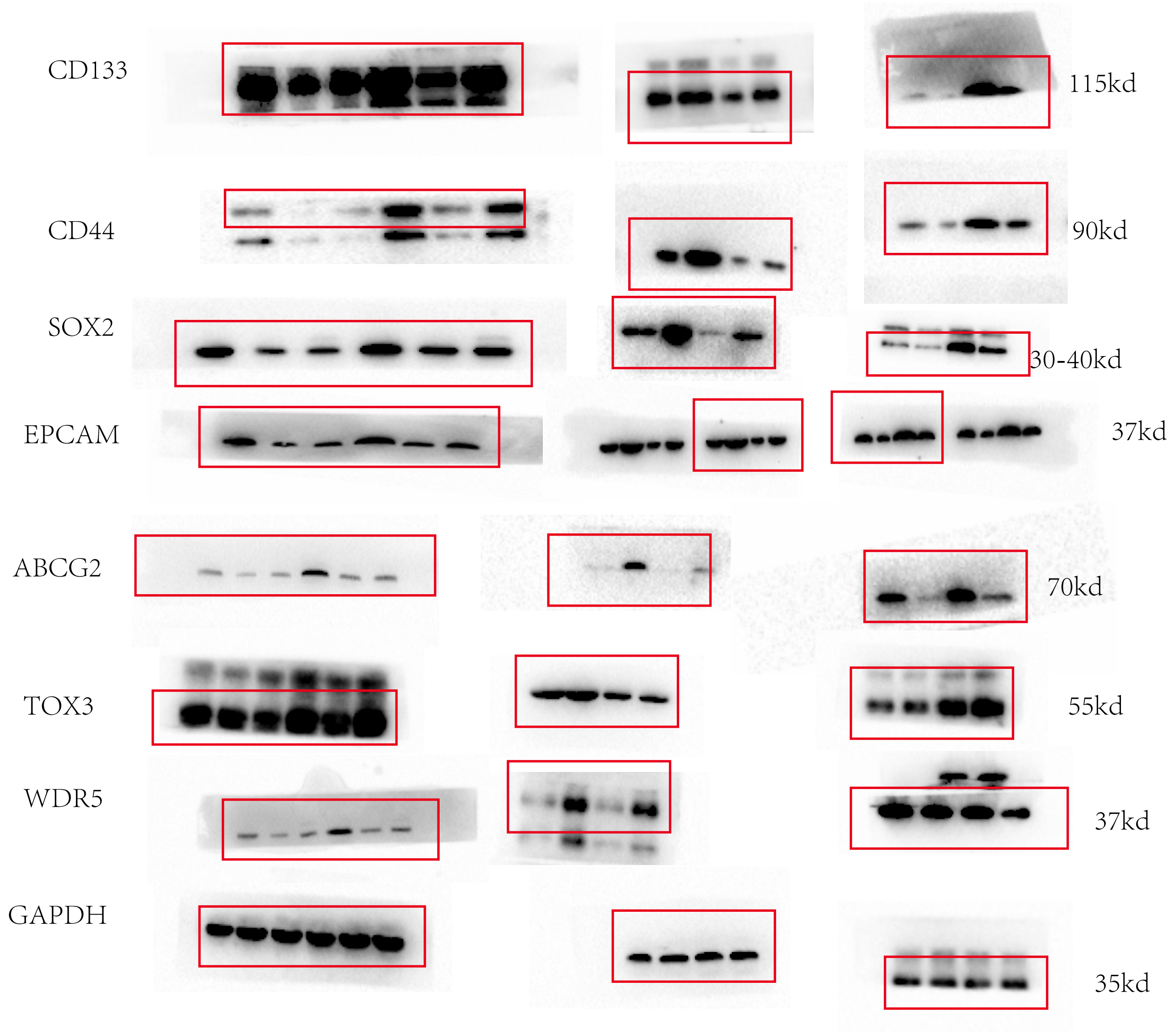

F5

F5B-LOVO

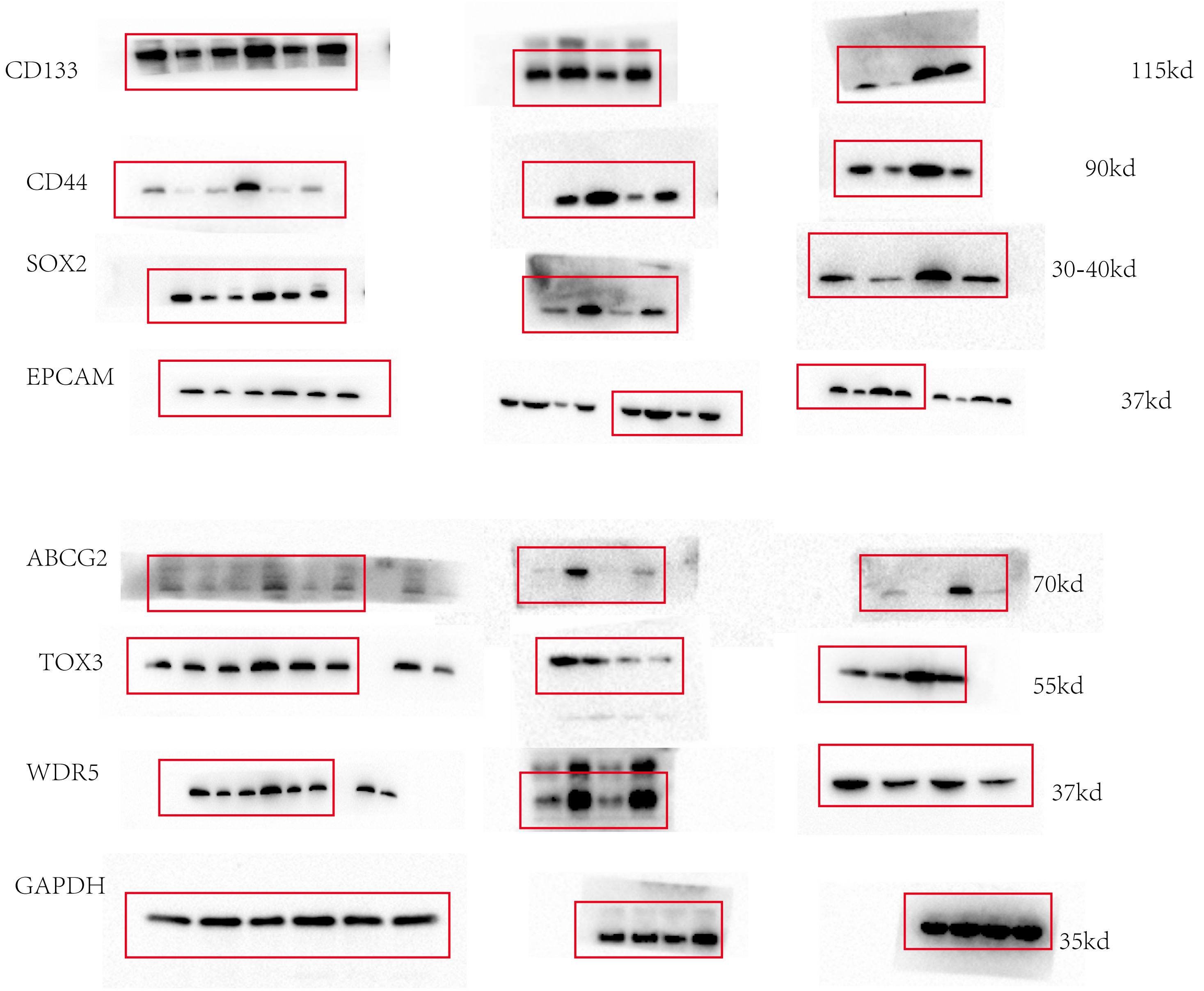
CD133
115kd
CD44
90kd
SOX2
30-40kd
EPCAM
37kd
ABCG2
70kd
TOX3
55kd
WDR5
37kd
GAPDH
35kd

F5

F5G

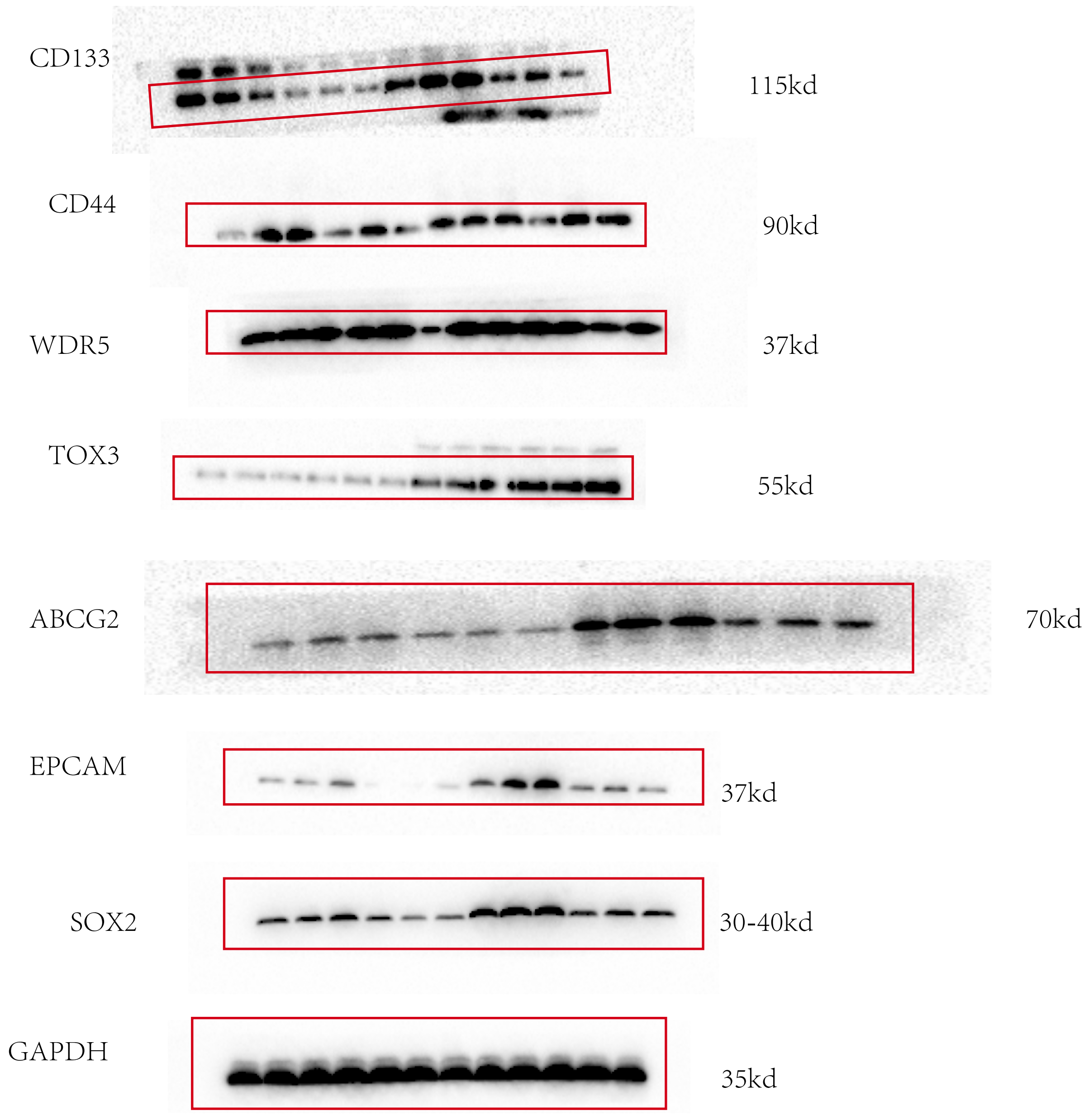

CD133
115kd
CD44
90kd
WDR5
37kd
TOX3
55kd
ABCG2
70kd
EPCAM
37kd
SOX2
30-40kd
GAPDH
35kd

F6

F6G

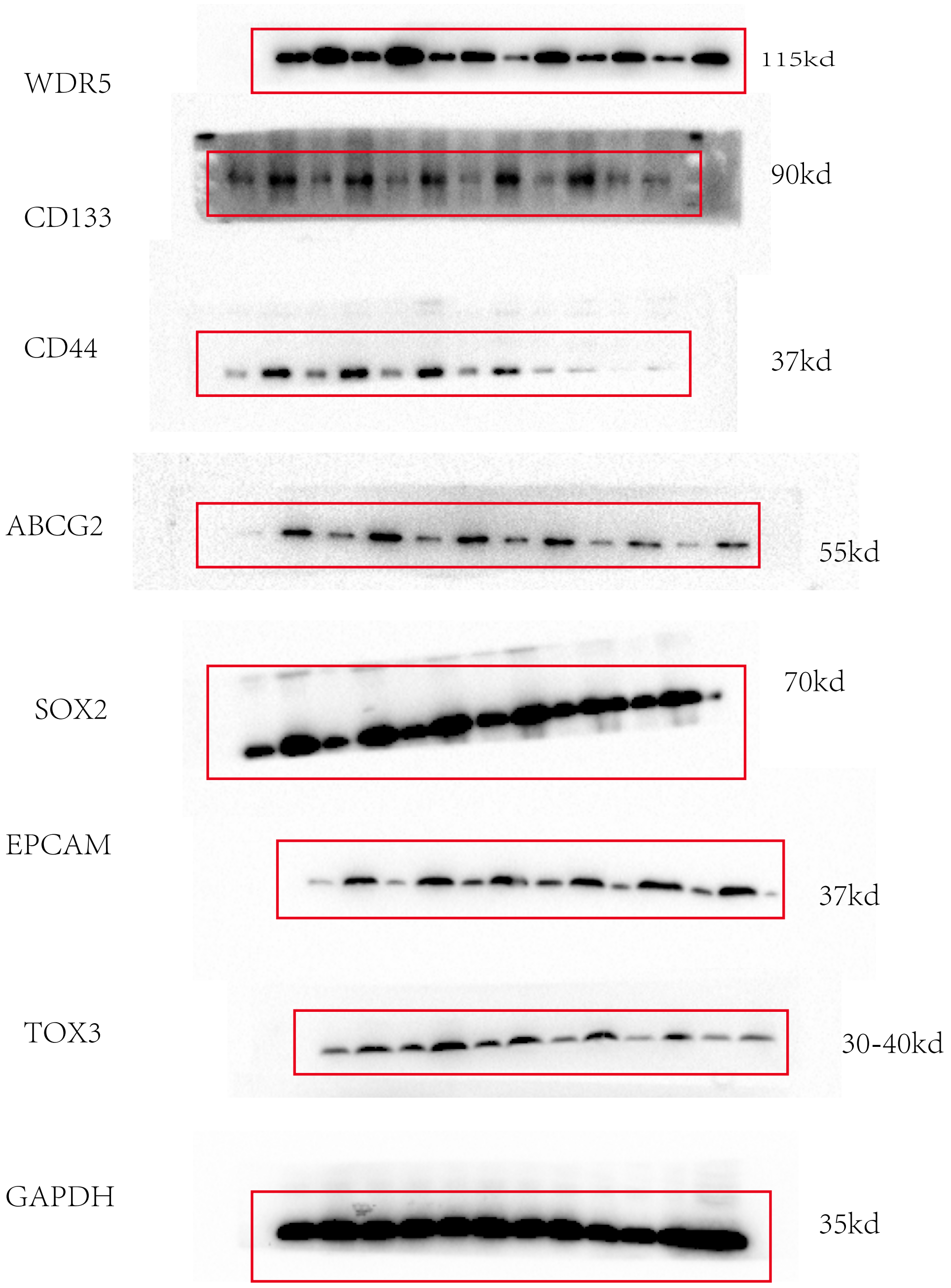
WDR5
115kd
CD133
90kd
CD44
37kd
ABCG2
55kd
SOX2
70kd
EPCAM
37kd
TOX3
30-40kd
GAPDH
35kd

SF1

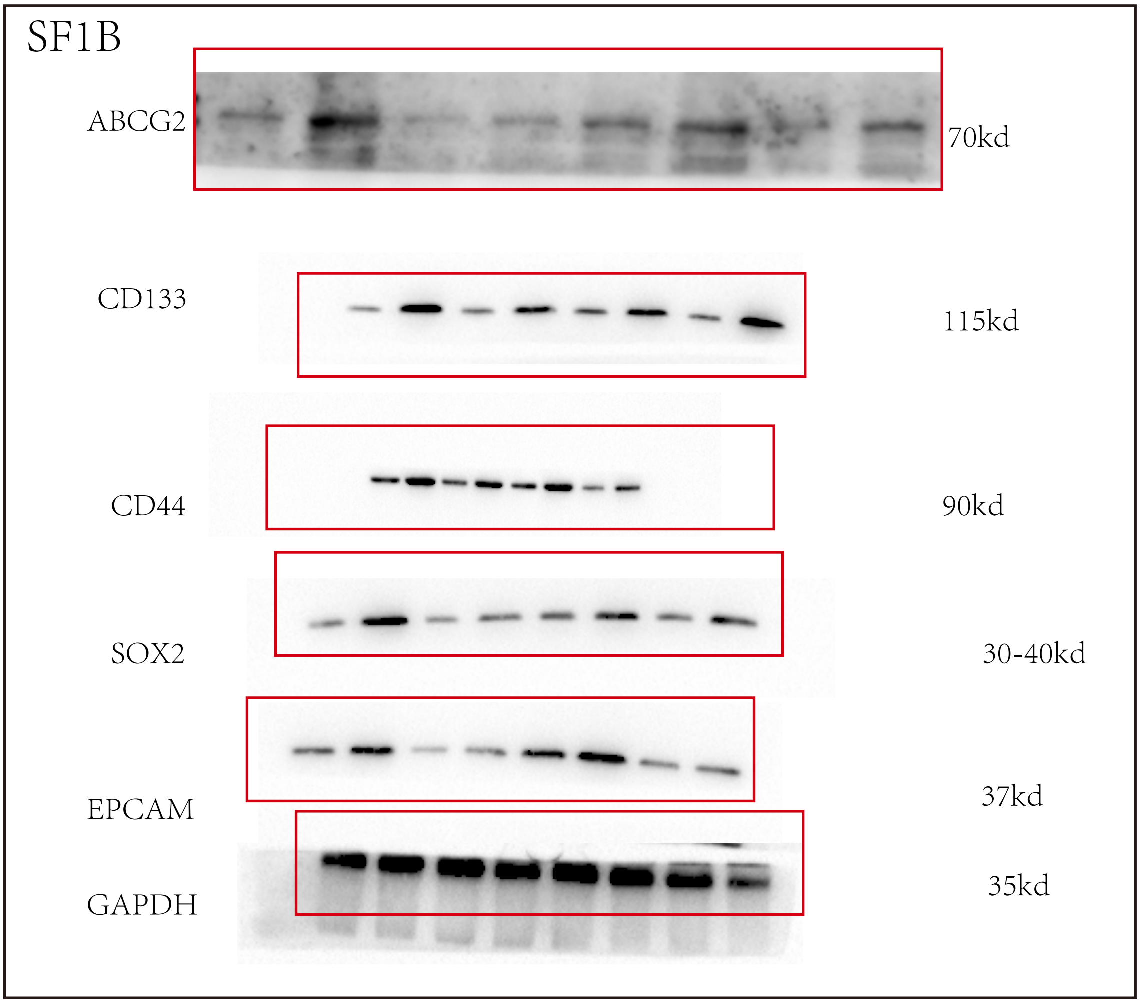
SF1B
ABCG2
70kd
CD133
115kd
CD44
90kd
SOX2
30-40kd
EPCAM
37kd
GAPDH
35kd

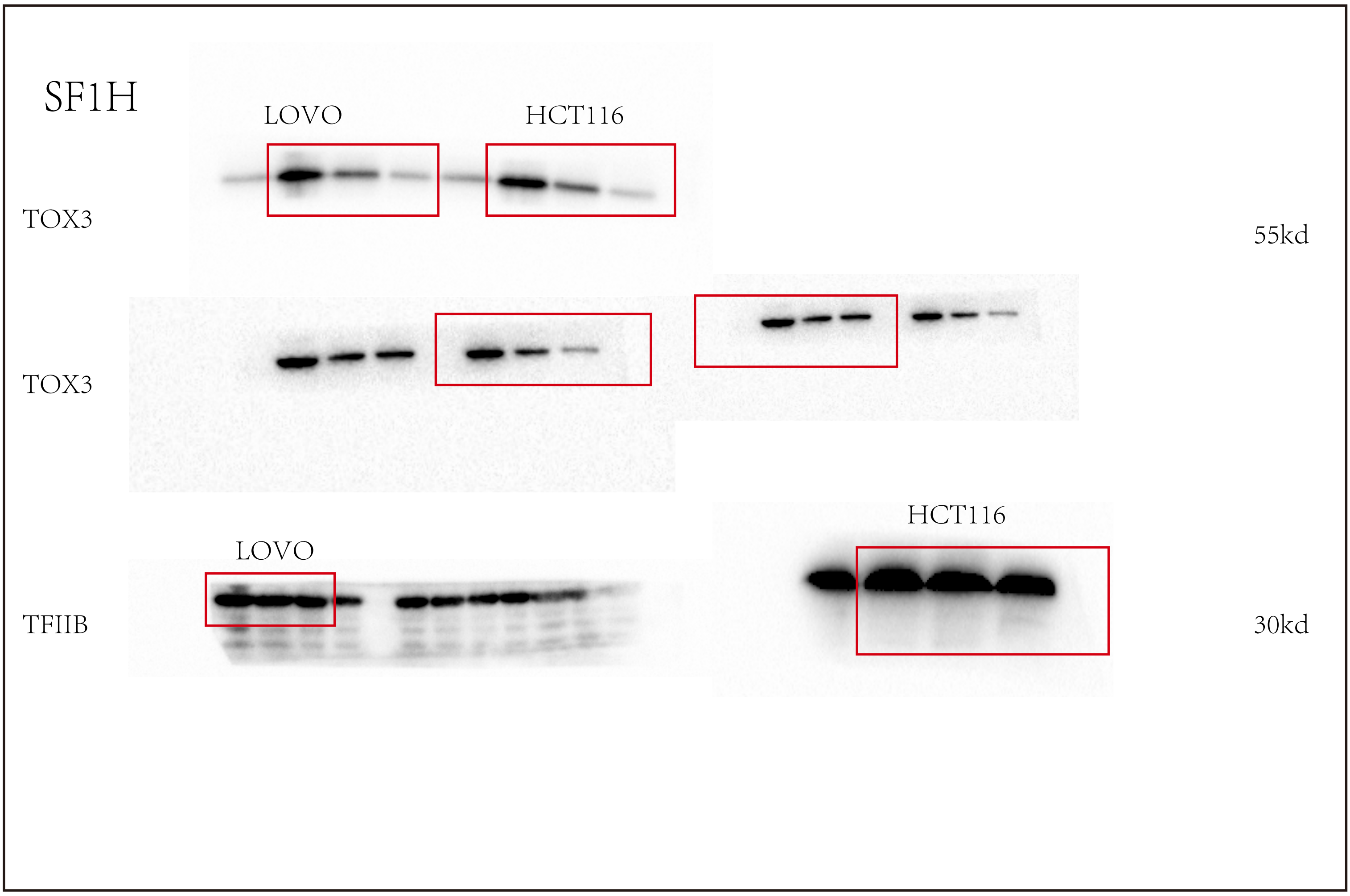
SF1H
LOVO
HCT116
TOX3
55kd
TOX3
LOVO
HCT116
TFIIB
30kd

SF2

SF2G-LOVO

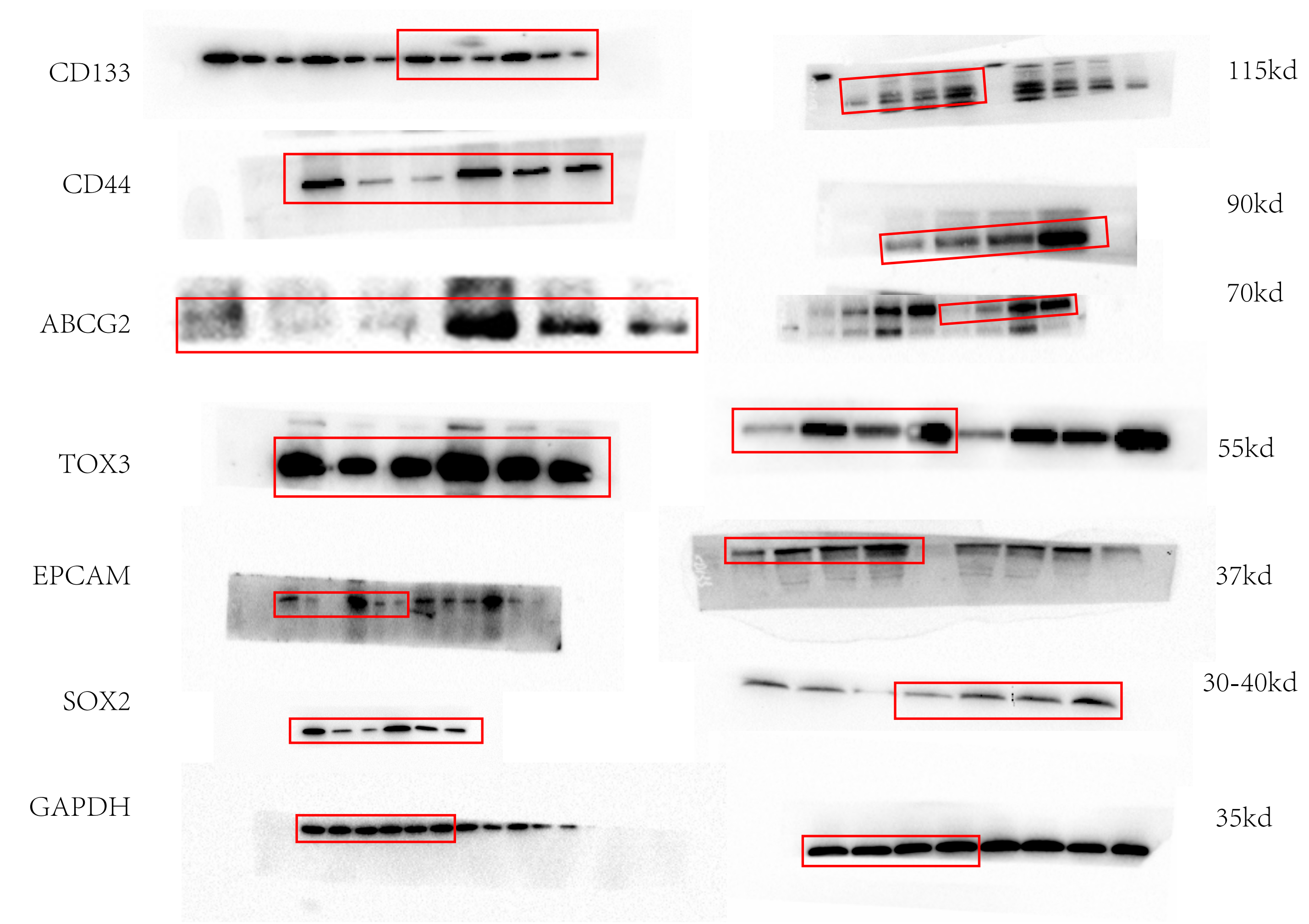

SF2G-HCT116

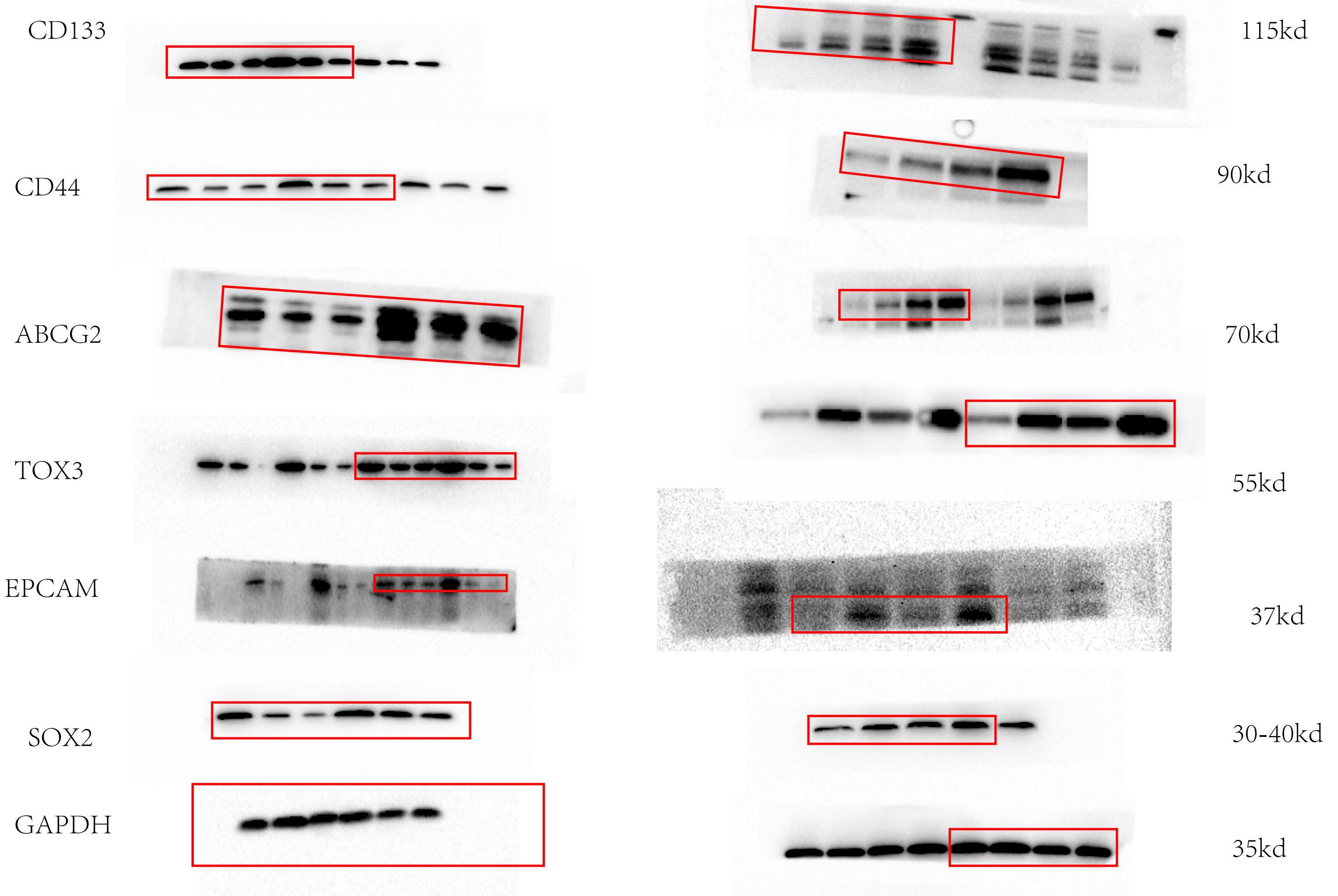

SF3

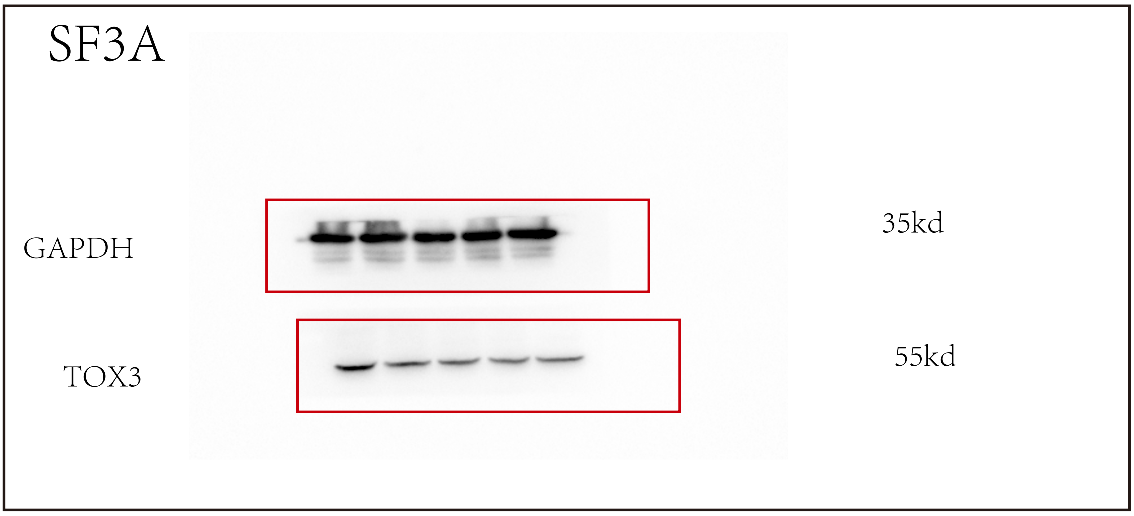
SF3A
GAPDH
35kd
TOX3
55kd

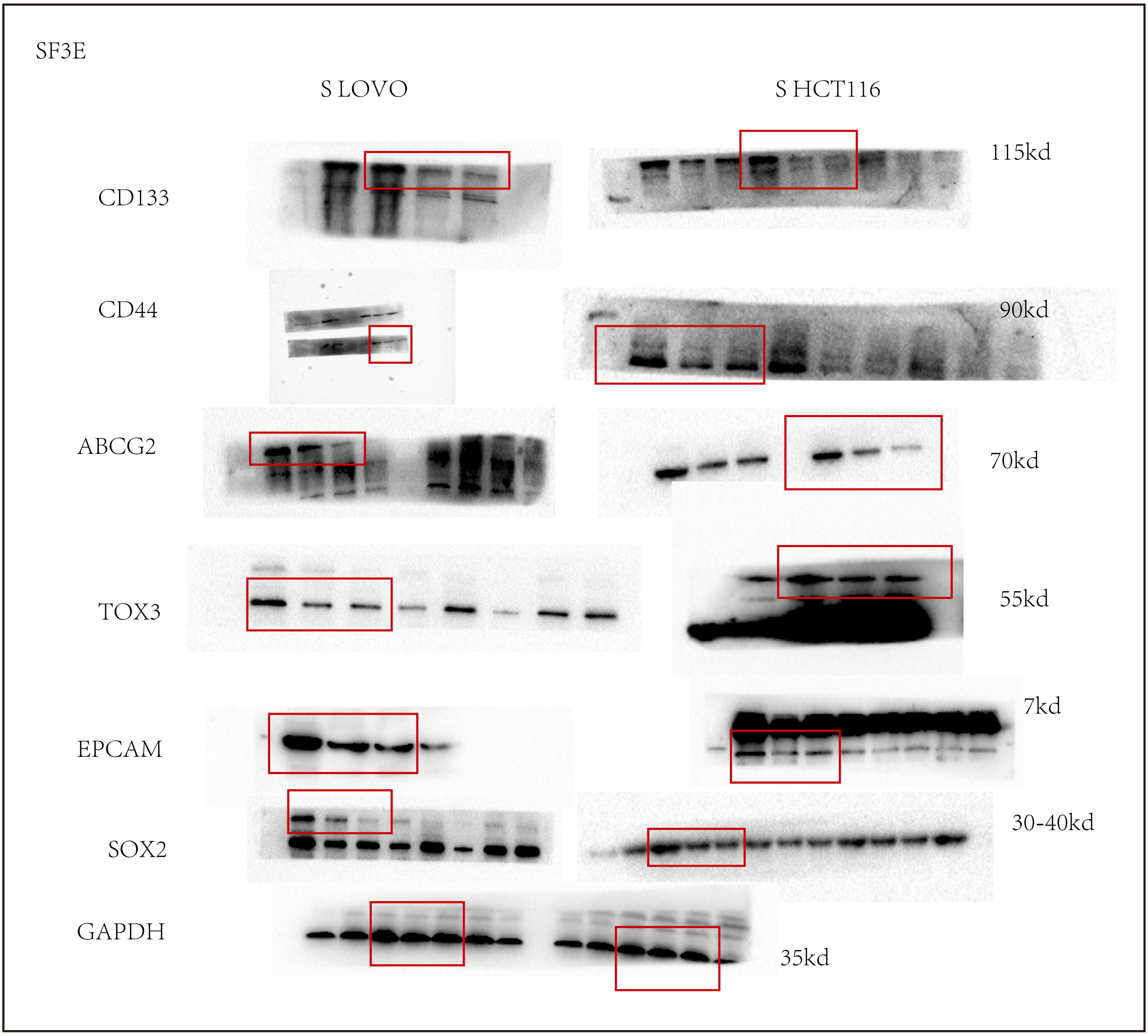
SF3E
S LOVO
S HCT116
CD133
115kd
CD44
90kd
ABCG2
70kd
TOX3
55kd
EPCAM
7kd
SOX2
30-40kd
GAPDH
35kd

SF6

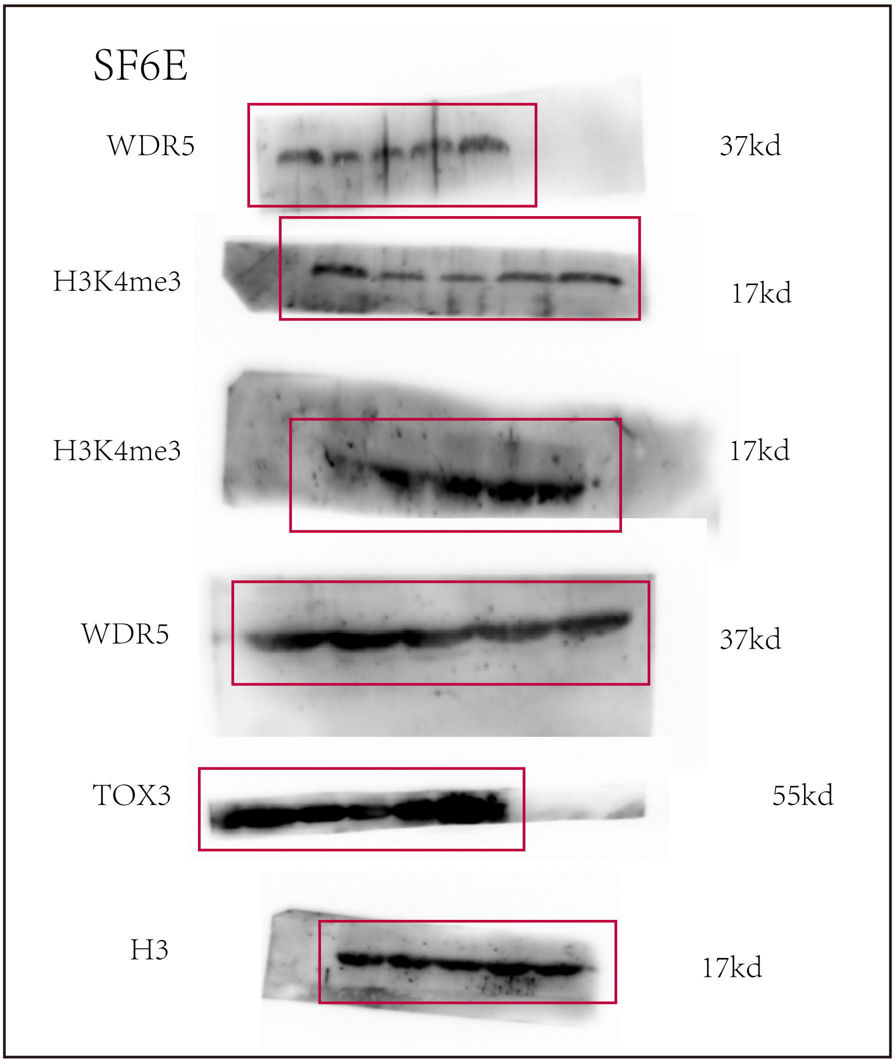
SF6E
WDR5
37kd
H3K4me3
17kd
H3K4me3
17kd
WDR5
37kd
TOX3
55kd
H3
17kd

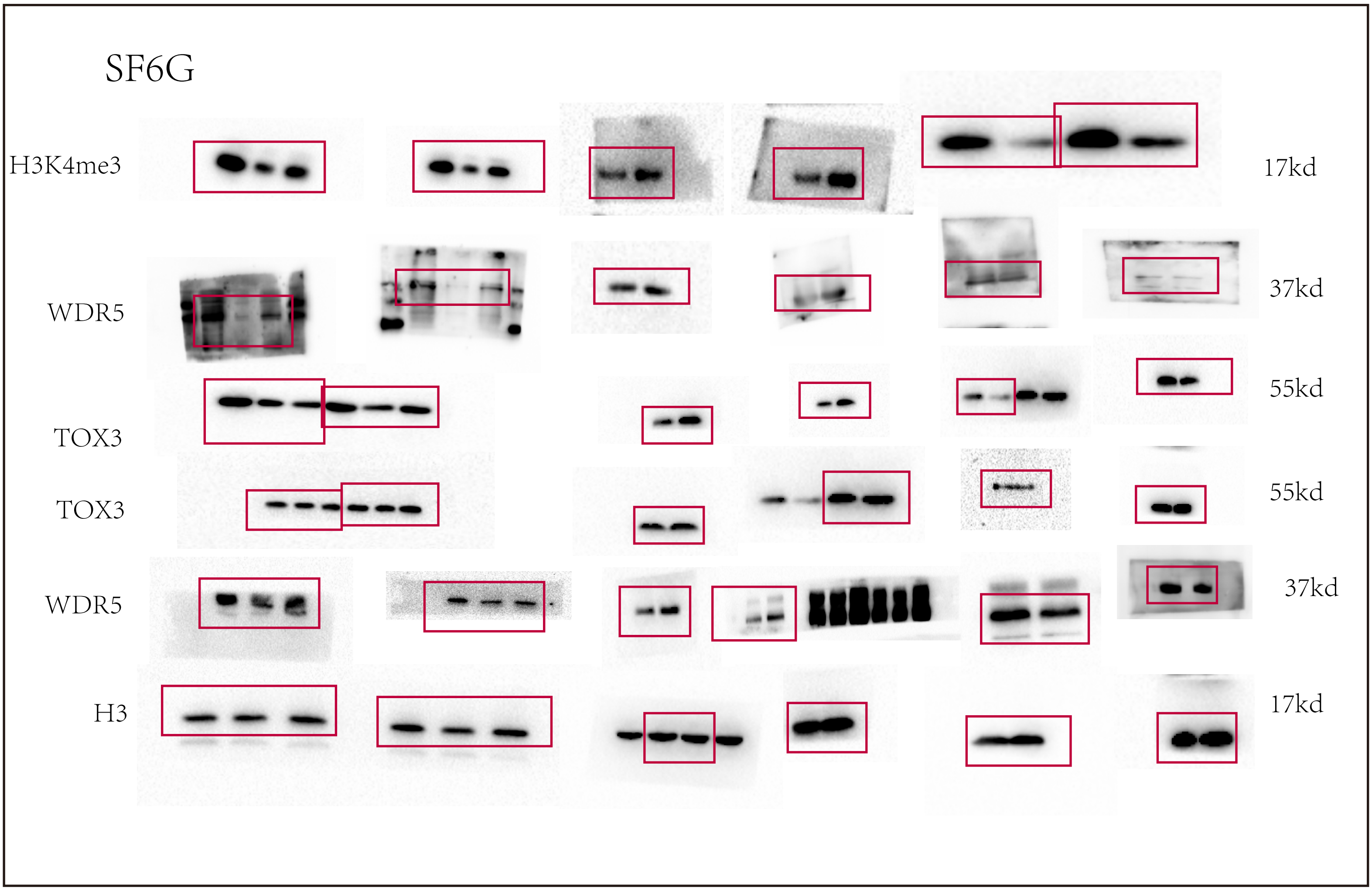
SF6G
H3K4me3
17kd
WDR5
37kd
TOX3
55kd
TOX3
55kd
WDR5
37kd
H3
17kd

SF7E

SF7E-sLOVO

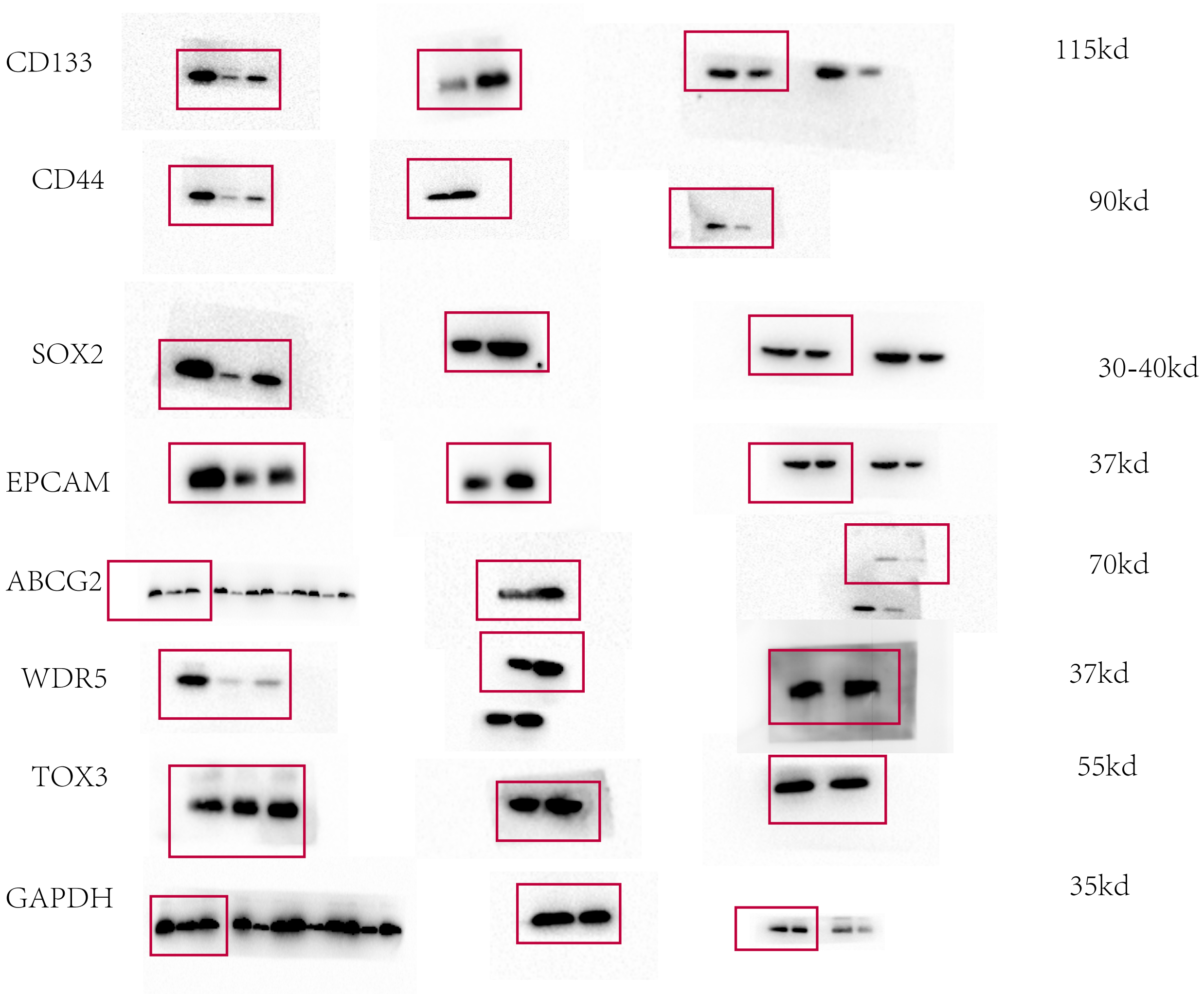

SF7E-sHCT116

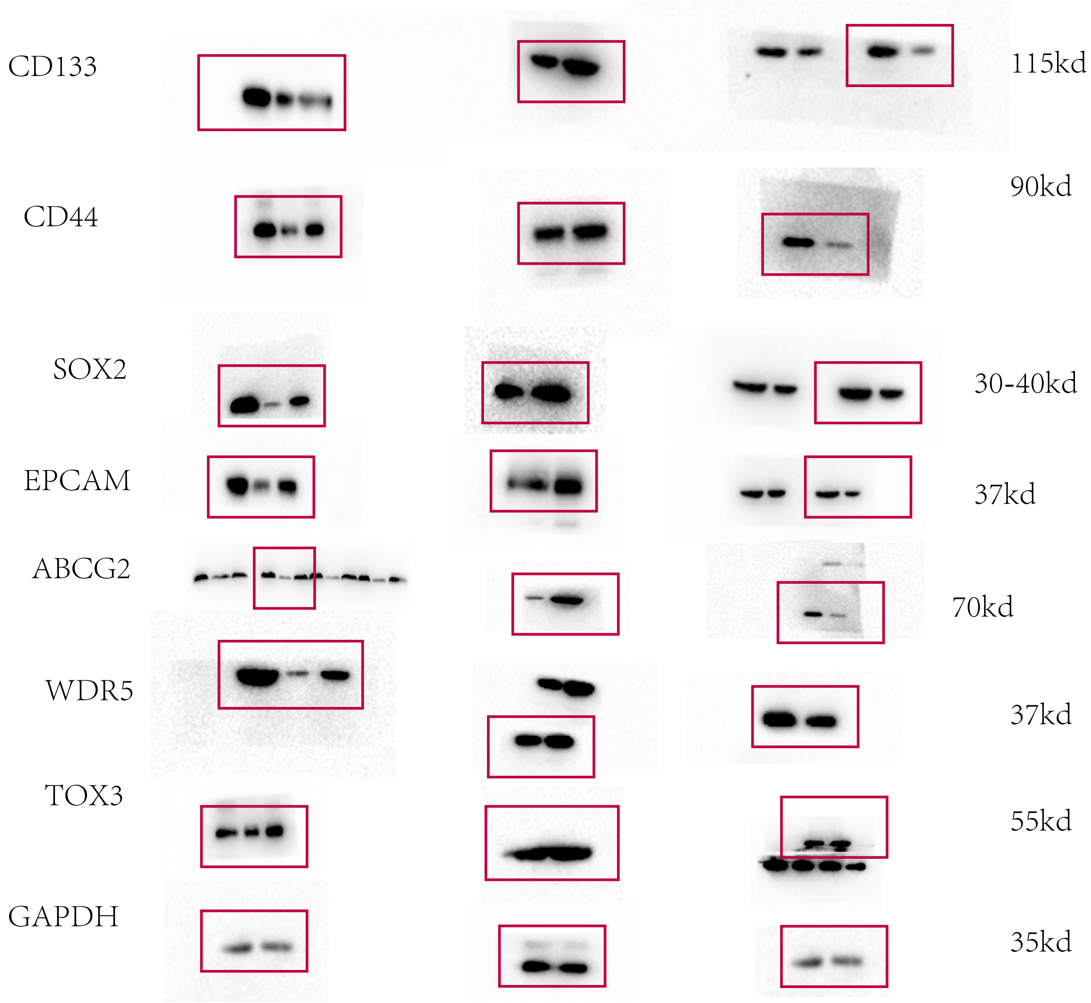

Supplement: S1 Raw Images — (PDF) [file pbio.3002256.s012.pdf]
