# Supplementary material for: A novel TOX3-WDR5-ABCG2 signaling axis regulates the progression of colorectal cancer by accelerating stem-like traits and chemoresistance
Source: PLoS Biol. 2023 Sep 14;21(9):e3002256. doi: 10.1371/journal.pbio.3002256 (PMC10501593; doi:10.1371/journal.pbio.3002256)
Supplement: S4 Table — (DOCX) [file pbio.3002256.s016.docx]

**Supplementary table 4. 5-year overall survival rate analysis by Kaplan-Meier for 94 patients with colorectal cancer**
